# Hyperglycemia Promotes Chemoresistance Through the Reduction of the Mitochondrial DNA Damage, the Bax/Bcl-2 and Bax/Bcl-XL Ratio, and the Cells in Sub-G1 Phase Due to Antitumoral Drugs Induced-Cytotoxicity in Human Colon Adenocarcinoma Cells

**DOI:** 10.3389/fphar.2018.00866

**Published:** 2018-08-13

**Authors:** Loredana Bergandi, Eleonora Mungo, Rosa Morone, Ornella Bosco, Barbara Rolando, Sophie Doublier

**Affiliations:** ^1^Department of Oncology, University of Turin, Turin, Italy; ^2^Department of Medical Sciences, University of Turin, Turin, Italy; ^3^Department of Drug Science and Technology, University of Turin, Turin, Italy

**Keywords:** hyperglycemia, chemoresistance, doxorubicin, 5-fluorouracil, cytotoxicity, cell damage, colon adenocarcinoma

## Abstract

Diabetes and cancer are common, chronic, and potentially fatal diseases that frequently co-exist. Observational studies clearly indicate that the risk of several types of cancer is increased in diabetic patients and a number of cancer types have shown a higher mortality rate in patients with hyperglycemic associated pathologies. This scenario could be due, at least in part, to a lower efficacy of the cancer treatments which needs to be better investigated. Here, we evaluated the effects of a prolonged exposure to high glucose (HG) to the response to chemotherapy on human colon adenocarcinoma HT29 and LOVO cell lines. We observed that hyperglycemia protected against the decreased cell viability and cytotoxicity and preserved from the mitochondrial DNA lesions induced by doxorubicin (DOX) and 5-fluorouracil (5-FU) treatments by lowering ROS production. In HT29 cells the amount of intracellular DOX and its nuclear localization were not modified by HG incubation in terms of Pgp, BCRP, MRP1, 5 and 8 activity and gene expression. On the contrary, in LOVO cells, the amount of intracellular DOX was significantly decreased after a bolus of DOX in HG condition and the expression and activity of MPR1 was increased, suggesting that HG promotes drug chemoresistance in both HT29 and LOVO cells, but in a different way. In both cell types, HG condition prevented the susceptibility to apoptosis by decreasing the ratio Bax/Bcl-2 and Bax/Bcl-XL and diminished the level of cytosolic cytochrome c and the cleavage of full length of PARP induced by DOX and 5-FU. Finally, hyperglycemia reduced cell death by decreasing the cell percentage in sub-G1 peak induced by DOX (via a cell cycle arrest in the G2/M phase) and 5-FU (via a cell cycle arrest in the S phase) in HT29 and LOVO cells. Taken together, our data showed that a prolonged exposure to HG protects human colon adenocarcinoma cells from the cytotoxic effects of two widely used chemotherapeutic drugs, impairing the effectiveness of the chemotherapy itself.

## Introduction

Antineoplastic drugs are an important therapeutic tool for cancer patients, but the development of multidrug resistance (MDR) may result in failure of the treatment, leading to tumor relapse and further progression. One of the mechanisms of MDR is the overexpression of the ATP binding cassette (ABC) transporters, such as P-glycoprotein (Pgp) and other MDR-related proteins (e.g., BCRP and MRPs), that actively extrude anticancer drugs, such as anthracyclines (e.g., doxorubicin, DOX), taxanes, Vinca alkaloids, epipodophyllotoxins, topotecan, mitomycin C (Comerford et al., 2002; Gottesman et al., 2002), leading to a decrease of the accumulation of intracellular chemotherapeutics and of their cytotoxic effects (Riganti et al., 2008, 2009; Doublier et al., 2012; Marin et al., 2016). Furthermore, MDR can develop by other numerous mechanisms including activation of detoxifying systems, changes in the expression/function of the molecular targets of anticancer drugs, enhanced ability of cancer cells to repair anticancer drug-induced DNA damage, and decreased and up-regulated expression/function of pro- or anti-apoptotic factors (Stattin et al., 2007).

The most recent International Diabetes Federation’s estimates indicate that 12.8% of adults – 382 million people worldwide in 2016 – have diabetes, and the number of people with the disease is set to rise beyond 592 million in less than 25 years, whereas 1.5 million people die from this disease every year (WHO, 2016). Hyperglycemia is often a consequence of a Western lifestyle that is associated with metabolic syndrome and type-2 diabetes or obesity, and high levels of glucose may be present in the blood for many years before the onset of overt diabetes (which may not appear at all throughout the life) (Atlas Website, 2014).

Hyperglycemia is a pathophysiological condition characterized by high blood glucose concentration that is not only a key pathological factor involved in diabetic complications (Nathan et al., 2013), but also it has been shown to predispose to cancer development (Biadgo et al., 2016) and progression (Duan et al., 2014). Indeed, multiple meta-analyses and large cohort studies support an association between hyperglycemia and increased cancer risk, especially for the breast, colorectal, pancreas, endometrium, and urinary tract cancer (Arcidiacono et al., 2012). It is becoming clear that sustained hyperglycemia is related not only to cancer incidence (Orgel and Mittelman, 2013), but also to cancer response to chemo-radiotherapy treatment (Meyerhardt et al., 2003; Caudle et al., 2008).

Even if several signaling pathways have been evaluated with respect to their involvement in drug resistance and hyperglycemia, gaining a better understanding of the mechanisms underlying the failure of cancer treatment may improve the drug efficacy in this condition.

In the present study, we evaluate the effects of a prolonged exposure to high glucose (HG) in the response to two chemotherapeutic drugs [DOX and 5-fluorouracil (5-FU)] on human colon adenocarcinoma (HT29 and LOVO cell lines).

## Materials and Methods

### Cells and Reagents

Human colon adenocarcinoma (HT29 and LOVO cell lines) obtained from the American Type Culture Collection (Rockville, MD, United States) were grown as a subconfluent monolayer in DMEM and F12/DMEM medium, respectively, containing 2 mM L-glutamine, 1% (v/v) antibiotic/antimycotic solution, and 10% (v/v) fetal bovine serum (FBS).

Unless otherwise specified, reagents were purchased from Sigma-Aldrich (Milan, Italy), whereas plastic ware was from Falcon (Becton Dickinson, Franklin Lakes, NJ, United States).

### Experimental Conditions

Different types of cells were cultured up to 12 weeks in medium containing normal glucose (control cells, G, 5.56 mM equal to 100 mg/dL) or HG (25 mM equal to 450 mg/dL). Mannitol (M, 25 mM) was used as osmotic control cells. Glucose concentrations of 5.56 mM and 25 mM, chosen in this study, correspond to glycemia in fasting healthy individuals and to hyperglycemia encountered in diabetic patients poorly controlled.

As preliminary experiments, we evaluated one-weekly lactate dehydrogenase (LDH) leakage and DOX intracellular accumulation up to 12 weeks in HG condition (data not shown). Given the similar results observed after 1 week and after the following experimental points, the experimental conditions have been standardized using cells cultured in very HG not less than 7 days (HG ≥ 7 days).

When required, cells were incubated for 24, 48, or 72 h before analysis with different doses of DOX or of 5-FU. 5-FU was chosen as one of the main elective drugs for colorectal cancer treatment, whereas DOX, used as drug for solid tumors, was also easily detectable in our experimental approaches.

### Cell Viability

Cells were grown in G and HG ≥ 7 days, treated for 24, 48, or 72 h, respectively, with chemotherapeutic agents at scalar concentrations (from 0.05 μmol/L to 10 μmol/L for DOX or from 1 μmol/L to 50 μmol/L for 5-FU, then stained for 1 h at 37°C in culture medium containing Neutral Red solution, washed three times with phosphate-buffered saline solution (PBS) and rinsed with stop buffer (1:1 of 4.02 g trisodium citrate in 153 mL H_2_O, 0.8 mL HCl 0.1 N in 86 mL H_2_O and 25 ml of 95% v/v methanol), as described (Gelsomino et al., 2013). The absorbance was read at 540 nm and the cell viability was evaluated by measuring the percentage of cells stained with neutral red dye, as reported previously (Repetto et al., 2008). The viability of untreated cells was considered 100%; the results were expressed as percentage of viable cells in each experimental condition versus untreated cells. The inhibitory concentration 50 (IC50), defined as the concentration of each drug able to decrease the cell viability by 50%, was calculated with the CompuSyn software^1^.

### Lactate Dehydrogenase (LDH) Leakage

For LDH leakage measurement, cells were grown in G and HG ≥ 7 days, then after 24 and 48 h of incubation with DOX (from 0.5 μmol/L to 10 μmol/L) or after 72 h of 5-FU (25 and 50 μmol/L), the extracellular medium was withdrawn and centrifuged at 12,000 × *g* for 30 min to remove cellular debris. Cells were washed with PBS, detached with trypsin/EDTA (0.05/0.02% v/v), resuspended in 1 ml of PBS and sonicated on ice with two 10-s bursts. LDH activity was measured in the extracellular medium and in the cell lysate, as previously described (Beutler, 1971; Riganti et al., 2006) to verify the cytotoxic effect of chemotherapeutic agents. Both intracellular and extracellular enzyme activities, measured spectrophotometrically as absorbance variation at 340 nm (37°C), were expressed as nmol NADH oxidized/min/dish; then extracellular LDH activity was calculated vs. the total (intracellular + extracellular) LDH activity in the dish.

### Measurement of Total Cellular and Mitochondrial ROS Production

Cells were grown in G and HG ≥ 7 days, then, after 24 h incubation in the absence or presence of 5 and 10 μM DOX or 25 and 50 μM 5-FU, cells were divided into two aliquots: one aliquot, to detect total cellular ROS production, has been resuspended in fresh appropriate medium; the other one, to detect mitochondrial ROS production, has been used to prepare mitochondrial fraction (see details in “Materials and Methods,” section “Cytochrome c Western Blot Analysis”). Total and mitochondrial lysates were loaded for 15 min with 10 μM 2′,7′-dichlorodihydrofluorescein diacetate (DCFH-DA). DCFH-DA is a cell-permeable probe that is cleaved intracellularly by non-specific esterases to form DCFH, which is further oxidized by ROS to form the fluorescent compound dichlorofluorescein (DCF). After the incubation, cells were washed twice with PBS to remove excess probe, and total and mitochondrial DCF fluorescence were determined at an excitation wavelength of 504 nm and an emission wavelength of 529 nm, using a Packard EL340 microplate reader (Bio-Tek Instruments, Winooski, VT, United States). The fluorescence value was normalized to the protein content and expressed as RFU/mg cellular proteins or mitochondrial proteins (Bergandi et al., 2010).

### Mitochondrial DNA (mtDNA) Damage Analysis

Cells were grown in G and HG ≥ 7 days, then incubated with DOX (5 and 10 μM for 24 h) or 5-FU (25 and 50 μM for 72 h) and washed with ice-cold PBS twice. Total DNA was purified using the Extract-NAmp^TM^ polymerase chain reactions from tissue kit containing all the reagents needed to rapidly extract and amplify human genomic DNA. Briefly, 10 μl of cells was mixed with 20 μl of the extraction solution and the mixture was incubated at room temperature for 5 min. After adding 180 μl of the neutralization solution, the extract was ready for determine the mtDNA integrity by qRT-PCR and the lesion rate by semi-long run rt-PCR (SLR rt-PCR).

The qRT-PCR was carried out as described by Yakes and Van Houten (1997) and Yang et al. (2014) amplifying equal amounts of total DNA isolated from differentially treated cells, employing 16.2 kb mitochondrial and 10.4 kb nuclear DNA fragments: 16.2.F 5′-TGAGGCCAAATATCATTCTGAGGGGC-3′, 16.2.R 5′-TTTCATCATGCGGAGATGTTGGATGG-3′; 10.4.F 5′-TGGGATTACACGTGTGAACCAACC-3′, 10.4.R 5′-GCTCTACCCTCTCCTCTACCGRCC-3′. Quantitative PCR amplification of a 10.4 kb fragment from the nuclear globin gene and a 16.2 kb fragment from the mitochondrial genome was used to detect mtDNA integrity.

The SLR rt-PCR amplifications were conducted in according to Rothfuss et al. (2010) in CFX96 Touch^TM^ Real-Time PCR Detection System (Bio-Rad, Hercules, CA, United States), and the amplification was monitored and analyzed by measuring the intercalation of the fluorescent dye to double-stranded DNA supplied by iTaq^TM^ Universal SYBR^®^ Green Supermix (Bio-Rad) according to the manufacturer’s instructions.

To compare the levels of DNA lesion in each tested region of the mitochondrial genome, two mtDNA fragments of different lengths [long fragments (L) ranging from 972 to 1037 bp and small fragments (S) from 54 to 87 bp, respectively], located in the same mitochondrial genomic region were used. To quantify the mitochondrial lesion frequency the four 1 kb sized mtDNA regions selected for the SLR rt-PCR approach were the following [(a) D-loop region, chrM:16021+423, (b) ATPase region, chrM:8204+9203, (c) ND4/5 region, chrM:12050+13049, (d) ND1/2 region, chrM:3962+4998]. The specific oligonucleotides were: for short amplicon AS1.F/R (5′-CCCTAACACCAGCCTAACCA-3′ and 3′-AAAGTGCATACCGCCAAAAG-5′), BS1.F/R (5′-CATGCCCATCGTCCTAGAAT-3′ and: 3′-ACGGGCCCTATTTCAAAGAT-5′), CS1.F/R (5′-TCCAACTCATGAGACCCACA-3′ and 3′-TGAGGCTTGGATTAGCGTTT-5′), DS1.F/R (5′-ACTACAACCCTTCGCTGACG-3′ and 3′-GCGGTGATGTAGAGGGTGAT-5′) and for long amplicon AL4.F/AS1.R (5′-CTGTTCTTTCATGGGGAAGC-3′ and 3′-AAAGTGCATACCGCCAAAAG-5′), BL1.F/R (5′-CATGCCCATCGTCCTAGAAT-3′ and 3′-TGTTGTCGTGCAGGTAGAGG-5′), CL1.F/R (5′-CACACGAGAAAACACCCTCA-3′ and 3′-CTATGGCTGAGGGGAGTCAG-5′), DL1.F/R (5′-CCCTTCGCCCTATTCTTCAT-3′ and 3′-GCGTAGCTGGGTTTGGTTTA-5′). The reaction mix consisted of 1 μl iTaq^TM^ Universal SYBR^®^ Green Supermix, 500 nM each forward and reverse primer (specific for the long and the short amplicon) and the equivalent quantities of template DNA (3 ng of total DNA) in 10 μl total volume. The cycling conditions include a pre-incubation phase of 10 min at 95°C followed by 40 cycles of 10 s 95°C, 10 s 60°C, and 10 s 72°C (for small fragments) or 50 s 72°C (for large fragments). Each sample was assayed in triplicate, fluorescence was continuously monitored versus cycle numbers and crossing point values were calculated by the CFX96 Touch^TM^ software (Bio-Rad). The PCR conditions for the different fragments were optimized to achieve similar amplification efficiencies required to compare different amplicons. The product specificity was monitored by melting curve analysis and product size was visualized on agarose gel by electrophoresis (data not shown).

For the quantification of damage in each mtDNA region, the crossing point (Cp) of isolated mitochondrial DNA from untreated sample was taken as reference. For each of the four mtDNA regions the difference in the crossing point ΔCp (long fragment/small fragment) was used as a measure of the relative mitochondrial lesion frequency with the 2^-ΔΔCT^ method in correlation to the amplification size of the long fragment and expressed as lesion per 10 kb DNA of each mtDNA region by including the size of the respective long fragment and displayed as average of at least three independent experiments (Rothfuss et al., 2010).

Lesion rate [Lesion per 10 kb DNA] = (1–2 - (Δ long - Δ short)) × 10000 [bp]/size of long fragment [bp].

### Doxorubicin Accumulation

Before every test, cells were grown in G and HG ≥ 7 days and incubated with DOX (5 and 10 μM for 24 h or 0.5 and 1 μM for 48 h), then washed twice in PBS, and cells were scrapered and collected. Cells were centrifuged for 30 s at 13,000 rpm (4°C) and resuspended in 700 μl of a 1:1 mixture of ethanol/0.3 N HCl. Fifty microliters of cell suspension were sonicated on crushed ice with two 10-s bursts (Labsonic sonicator, 100 W) and used for measurement of cellular proteins; the remaining part was checked for the DOX content using a Perkin-Elmer LS-5 spectrofluorimeter (Perkin Elmer). Excitation and emission wavelengths were 475 and 553 nm, respectively. A blank was prepared in the absence of cells in every set of experiments and its fluorescence was subtracted from that obtained in the presence of cells (Riganti et al., 2011). Fluorescence was converted in nmol of DOX per milligram of cellular proteins, using a calibration curve prepared previously.

### Preparation of Samples for RP-HPLC Quantification of Doxorubicin and 5-Fluorouracil and Their Metabolites

Cells were grown in G and HG ≥ 7 days and then incubated with DOX (5 and 10 μM for 24 h) washed twice in ice-cold PBS and resuspended in 500 μl of PBS. Cellular samples derived from DOX incubation were diluted 1:1 with acetonitrile 0.1% HCOOH; the mixture was sonicated, centrifuged for 10 min at 2150 g, filtered (0.45 μm PTFE) and analyzed by RP-HPLC. HPLC analyses were performed with a HP 1200 chromatograph system (Agilent Technologies, Palo Alto, CA, United States) equipped with a quaternary pump (model G1311A), a membrane degasser (G1322A), a multiple wavelength UV-vis detector (MWD, model G1365D), a thermostated column compartment (model G1316A), and fluorescence detector (FLD, model G1321A) integrated in the HP1200 system. Data analysis was performed using a HP ChemStation system (Agilent Technologies). Samples were analyzed according to Sakai-Kato et al. (2010) on a Tracer Excel 120 ODSB column (25 × 0.46, 5 μm) (Teknokroma). The injection volume was 20 μL (Rheodyne, Cotati, CA, United States). The mobile phase consisting of acetonitrile (A) and 0.1% HCOOH (B) at flow-rate = 1.0 mL/min with gradient conditions: 35% A until 5 min, from 35 to 80% A between 5 and 10 min, 80% A between 10 and 20 min, and from 80 to 35% A between 20 and 25 min. The column effluent was monitored by MWD at 234 and 480 nm referenced against a 800 nm wavelength. Data analysis was performed with Agilent ChemStation. This method simultaneously determines DOX and its main metabolites in HT29 and LOVO cells. The values obtained from integration of the peak of DOX were interpolated in a calibration curve obtained using standard solutions of DOX at 0.1 μM to 50 μM (*r*^2^ = 0.996).

The micromolar concentration value was normalized to the protein content and expressed as nmol DOX or metabolites/mg cell proteins.

Cellular samples derived from 5-FU incubation (25 and 50 μM for 24, 48, and 72 h) were added with acetonitrile (4:1, v/v); the mixture was sonicated, centrifuged for 10 min at 2150 × *g*, filtered (0.45 μm PTFE) and analyzed by liquid chromatography-electrospray ionization-tandem mass spectrometry (LC-ESI-MS) analyses. LC-ESI- MS analyses were performed with an Acquity Ultra Performance LC^TM^, Waters Corporation, Milford, MA, United States, equipped with BSM, SM, CM, and PDA detector. All the chromatographic separations were performed on Aquasil C18 column (20 × 0.46, 5 μm) (Thermo) as a stationary phase. Aliquots of 5 μL were injected onto the system and eluted with a mobile phase (flow rate, 0.5 mL/min) consisting of acetonitrile, and 0.1% HCOOH 5/95 v/v. The eluate was injected into the electrospray ion source (ESI), and monitored using Micromass Quattro micro^TM^ API ESCi multi-mode ionization Enabled as detector. MS spectra were acquired and processed using MassLynx software. The operating conditions on the triple quadruple mass spectrometer were as follows: negative mode; drying gas (nitrogen) heated at 350°C at a flow rate of 800 L/h; nebulizer gas (nitrogen) at 80 L/h; capillary voltage in positive mode at 3000 V; cone voltage at 40 V. The molecular ion [M-H]^-^ was employed for quantitative measurements of analyte. The values obtained from integration of the peak of compound were interpolated in a calibration curve obtained using standard solutions at 0.01–20 μM. The amount of compound was expressed as nmol/mg cell proteins.

5-FU content in cellular culture medium was assessed after 72 h incubation in the presence of HT29 and LOVO cells. Supernatant was diluted 1:1 with acetonitrile; the mixture was sonicated, centrifuged for 10 min at 2150 × *g*, filtered (0.45 μm PTFE) and analyzed by RP-HPLC. HPLC analyses were performed with a HP 1200 chromatograph system previously described.

Samples were analyzed according to Carli et al. (2009) on an Aquasil C18 column (20 × 0.46, 5 μm) (Thermo); the injection volume was 20 μL (Rheodyne, Cotati, CA, United States). Elution was performed under gradient conditions, employing a mobile phase consisting of acetonitrile (A) and 0.1% HCOOH (B): 0% A until 8 min, from 0 to 10% A between 8 and 15 min, 10% A between 15 and 20 min, and from 10 to 0% A between 20 and 25 min. The flow-rate was 1.0 ml/min and the UV detector was set at 270 nm referenced against a 800 nm wavelength. Data analysis was performed with Agilent ChemStation. The values obtained from integration of the peak of 5-FU and metabolites were interpolated in calibration curves obtained using standard solutions of the analytes at 0.1 μM to 50 μM (*r*^2^ = 0.995). The HPLC value was normalized to the protein content and expressed as nmol 5-FU/mg cell proteins.

### Immunofluorescence Staining

0.5 × 10^6^ cells, incubated in the experimental conditions reported previously, were grown on sterile glass coverslips, rinsed with PBS, fixed with 4% w/v paraformaldehyde (diluted in PBS) for 15 min, washed three times with PBS and incubated with 4′,6-diamidino-2-phenylindole dihydrochloride (DAPI, diluted 1: 20000) for 3 min at room temperature in the dark. Fluorescently labeled cells were washed three times with PBS and once with water, then the slides were mounted with 4 μl of Gel Mount Aqueous Mounting and examined with a Leica DC100 fluorescence microscope (Leica Microsystems GmbH, Wetzlar, Germany). For each experimental point, a minimum of five microscopic fields were examined.

### Pgp and MRP Activities

The efflux of rhodamine 123, a substrate of Pgp and MRP, was taken as an index of Pgp plus MRP activity. Cells were grown in G and HG ≥ 7 days, then washed with fresh PBS, detached with cell dissociation solution and resuspended at 5 × 10^5^ cells/mL in 1 mL of DMEM medium containing 5% FBS. The samples were maintained at 37°C for 20 min in the presence of 1 μg/mL rhodamine 123. After this incubation time, cells were washed and resuspended in 500 μl of PBS, and the intracellular rhodamine content, which is inversely related to its efflux, was analyzed for the rhodamine content, using a PerkinElmer LS-5 spectrofluorimeter. Excitation and emission wavelengths were 507 and 527 nm, respectively. An aliquot of sample was used for the determination of the intracellular proteins. A blank was prepared in the absence of cells in each set of experiments, and its fluorescence was subtracted from the one measured in each sample. Fluorescence was converted in % of rhodamine 123/mg of cell proteins using a calibration curve prepared previously.

### BCRP Activity

The efflux of Hoechst 33342, a specific substrate of BCRP, was taken as an index of BCRP activity. Cells were grown in G and HG ≥ 7 days, then washed with PBS and resuspended in 500 μL of DPBS buffer (129 mM NaCl, 2.5 mM KCl, 7.4 mM Na_2_HPO_4_, 1.3 mM KH_2_PO_4_, 1 mM CaCl_2_, 0.7 mM MgSO_4_, 5.3 mM glucose; pH 7.4), in the presence of 50 μM Hoechst 33342 for 15 min at 37°C. Then 400 μl of stop solution (210 mM KCl, 2 mM Hepes; pH 7.4) was added and cells were lysed with 100 μl of 0.1% v/v Triton-X 100, dissolved in 0.3% v/v NaOH.

An aliquot of sample was used for the determination of the intracellular proteins, and the remaining part was analyzed for the Hoechst content, using a PerkinElmer LS-5 spectrofluorimeter. Excitation and emission wavelengths were 370 and 450 nm, respectively. A blank was prepared in the absence of cells in each set of experiments, and its fluorescence was subtracted from the one measured in each sample. Fluorescence was converted in % of Hoechst/mg of cell proteins using a calibration curve prepared previously.

### Real-Time Polymerase Chain Reaction (qRT-PCR)

Before every test, cells were grown in G and HG ≥ 7 days and then washed with PBS. Total RNA was extracted with TRIzol^®^ (Invitrogen, Thermo Fisher Scientific, Waltham, MA, United States). One μg of total RNA were reversely transcribed into cDNA, in a final volume of 20 μl, using the iScript^TM^ cDNA Synthesis Kit (Bio-Rad, Hercules, CA, United States) according to the manufacturer’s instructions. The RT-PCR primers were designed with NCBI/Primer-BLAST. Quantitative PCR was carried out in a final volume of 20 μl using the iTaq^TM^ Universal SYBR^®^ Green Supermix (Bio-Rad, Hercules, CA, United States) with specific primers for the quantitation of *ATP-binding cassette, sub-family C* (CFTR/MRP) *member 1* (ABCC1) (MRP1, 5′-TCTGGTCAGCCCAACTCTCT-3′ and 5′-CCTGTGATCCACCAGAAGGT-3′), *ATP-binding cassette, sub-family C* (CFTR/MRP) *member 5* (ABCC5) (MRP5, 5′-CCCAGGCAACAGAGTCTAACC-3′ and 5′-CGGTAATTCAATGCCCAAGTC-3′), *ATP-binding cassette, sub-family C* (CFTR/MRP) *member 8* (ABCC8) (MRP8, 5′-TCTGCGACCTTCTTGTTTGG-3′ and 5′-TCAGTACAGCATTTGCAACACTT-3′), *ATP-binding cassette, sub-family G ABCG2* (BCRP, 5′-AGCTGCAAGGAAAGATCCAA-3′ and 5′-TCCAGACACACCACGGATAA-3′), *ATP-binding cassette, sub-family B* (MDR/TAP) *member 1* (ABCB1) (Pgp, 5′-GACTGAGCCTGGAGGTGAAG-3′ and 5′-CCACCAGAGAGCTGAGTTCC-3′), *topoisomerase II alpha* (TOPO II alpha, 5′-GCCCTCAAGAAGATGGTGTG-3′ and 5′-TGCCAATGTAGTTTGTTTCTTG-3′) and *beta 2-microglobulin* (β2M, 5′-AGCAAGGACTGGTCTTTCTATCTC-3′ and 5′-ATGTCTCGATCCCACTTAACTA-3′) genes.

PCR amplification was 1 cycle of denaturation at 95°C for 30 s, 40 cycles of amplification including denaturation at 95°C for 30 s and annealing/extension at 60°C for 1 min. Standard curves, with serially diluted solutions (1:1; 1:10; 1:100; and 1:1000 for MRP1/5/8 and BCRP genes and 1:1; 1:10; 1:100 for Pgp) of cDNAs obtained as a template for each gene, were included in each PCR and amplified by target-specific primer sequence to quantify the PCR baseline subtracted relative fluorescence unit. The threshold cycle (Ct) reflects the cycle number at which the fluorescence generated within a reaction crosses the threshold line. The quantification of each sample was performed comparing each PCR gene product with B2M, used as reference gene to normalize the cDNA in different samples, and expressed in arbitrary units, using the Bio-Rad Software Gene Expression Quantitation (Bio-Rad Laboratories), calculated using the 2^-ΔΔCT^ method (Livak and Schmittgen, 2001). Analyzed transcripts exhibited high linearity amplification plots (*r* > 0.98) and similar PCR efficiency (84% for MRP1, 83.4% for MRP5, 85.6% for MRP8, 95% for BCRP, 83.8% for Pgp, 92% for TOPO II alpha, and 94% for β2M), confirming that the expression of each gene could be directly compared. The specificity of PCRs was confirmed by melt curve analysis. Non-specific amplifications were never detected.

### Bcl-2, Bcl-XL, Bax, PARP Western Blot Analysis

Cells were grown in G and HG ≥ 7 days and then incubated with DOX (5 and 10 μM for 24 h) or 5-FU (25 and 50 μM for 72 h). Cells were collected and washed twice in PBS, then lysed for 1 h in ice-cold lysis buffer (50 mM Tris–HCl, 150 mM NaCl, 5 mM EDTA, pH 7.4 supplemented with the protease inhibitor cocktail set III (Sigma-Aldrich, Milan, Italy), 1 mM sodium orthovanadate, 1 mM phenylmethanesulfonyl fluoride, 1 mg/ml aprotinin, 50 mM sodium fluoride, 1% Triton X-100). Cell lysates were then centrifuged for 15 min at 13,000 rpm at 4°C (Poornima et al., 2014). Proteins were subjected to SDS-PAGE and subsequently transferred to PVDF membrane. The blots were blocked with 5% non-fat milk in PBS at RT for 1 h and incubated overnight with the following antibodies: mouse anti-Bcl-2 antibody (1–2 μg/ml in PBS-BSA 1%, from Thermo Fisher Scientific, Waltham, MA, United States), mouse anti-Bcl-XL antibody (diluted 1:250 in PBS-BSA 1%, from Thermo Fisher Scientific), mouse anti-Bax antibody (diluted 1:100 in PBS-BSA 1%, from Thermo Fisher Scientific), mouse anti-Poly-(ADP-Ribose)-Polymerase full length antibody (diluted 1:500 in PBS-BSA 1%, from Roche, Basel, Swiss) and mouse anti-GAPDH antibody (diluted 1:1000 in PBS-BSA 1% from Sigma-Aldrich, Italy). The latter antibody was used to check the equal protein loading in extracts. After an overnight incubation, the membrane was washed with 0.1% v/v PBS-Tween and subjected for 1 h to a peroxidase-conjugated anti-mouse secondary antibody (diluted 1:5000 in 5% w/v PBS-Tween with milk, Bio-Rad Laboratories, Hercules, CA, United States). The membrane was washed again with PBS-Tween, and proteins were detected and quantified by ChemiDoc^TM^ MP System (Bio-Rad Laboratories, Hercules, CA, United States). Densitometric analysis was carried out using ImageJ software^2^.

### Cytochrome c Western Blot Analysis

Cells were grown in G and HG ≥ 7 days and then incubated with DOX (5 and 10 μM for 24 h) or 5-FU (25 and 50 μM for 72 h). The cell supernatant was collected, centrifuged at 4,000 rpm for 2 min at 4°C and, after washing with ice-cold PBS twice, resuspended in mitochondrial lysis buffer A (50 mM Tris, 100 mM KCl, 5 mM MgCl_2_, 1 mM EDTA, ATP 1.8 mM, pH = 7.2). 5 × 10^6^ cells were washed twice in ice-cold PBS, then added to the pellet derived from cell supernatant and resuspended in 500 μl of mitochondrial lysis buffer A, supplemented with the protease inhibitor cocktail set III (Sigma-Aldrich, Milan, Italy), 1 mM phenylmethylsulfonyl fluoride and 2.5 mM sodium fluoride. Samples were clarified by centrifuging at 2,000 rpm for 2 min at 4°C, and the supernatant was collected and centrifuged at 13,000 rpm for 5 min at 4°C. The supernatant (cytosolic fraction) was aliquoted and the pellet containing mitochondria (mitochondrial fraction) was washed in 500 μl buffer A and resuspended in 250 μl mitochondrial resuspension buffer B (250 mM sucrose, 15 mM K_2_HPO_4_, 2 mM MgCl2, 0.5 mM EDTA, 5% w/v BSA). The aliquot of mitochondrial fraction was sonicated, used for the measurement of protein concentration and stored at -80°C until the use. Ten micrograms from mitochondrial extracts were subjected to 15% SDS-PAGE, transferred to PVDF membrane and probed with the following antibodies: mouse anti-cytochrome c antibody (diluted 1:250 in PBS-BSA 1%, from BD Biosciences, San Jose, CA, United States) and mouse anti-β-tubulin (diluted 1:500 in PBS-BSA 1% from Santa Cruz Biotechnology Inc., Santa Cruz, CA, United States). The latter antibody was used to check the equal protein loading in mitochondrial extracts. After an overnight incubation, the membrane was washed with 0.1% v/v PBS-Tween and subjected for 1 h to a peroxidase-conjugated anti-mouse secondary antibody (diluted 1:5000 in 5% w/v PBS-Tween with milk, Bio-Rad Laboratories, Hercules, CA, United States). The membrane was washed again with PBS-Tween, and proteins were detected and quantified by ChemiDoc^TM^ MP System (Bio-Rad Laboratories, Hercules, CA, United States). Densitometric analysis was carried out using ImageJ software (see footnote 2).

### Cell Cycle Analysis

Cells were grown in G and HG ≥ 7 days, then, after 24 h incubation in the absence or presence of 5 and 10 μM DOX or after 72 h with 25 and 50 μM 5-FU, cells were collected, fixed in 70% cold ethanol for 30 min on ice and centrifuged at 1,800 rpm for 10 min. Cells were washed with PBS, then 1 × 10^6^ cells/ml were resuspended in 1 ml PBS and incubated in propidium iodide solution (20 μg/ml propidium iodide and 0.2 mg/ml RNAseA in PBS) for 1 h at room temperature. Cellular DNA content was analyzed by Coulter EPICS XL (CoulterCorp, Hialeah, FL, United States) flow cytometry and to perform the analysis only on intact single cells, an electronic gate for doublet and clump exclusion was used (Lanza et al., 1996; Catalano et al., 2009). To quantify the relative proportions of the various cell cycle phases, a Multicycle software (Phoenix Flow Systems, San Diego, CA, United States) was used.

### Topoisomerase II Alpha Assay

The *in vitro* activity of topoisomerase II alpha was measured using the Topoisomerase II Assay Kit plasmid based (Topogen Inc., Port Orange, FL, United States), following the manufacturer’s instructions.

Cells were grown in G and HG ≥ 7 days, then incubated with DOX 5 and 10 μM for 24 h and washed with ice-cold PBS twice. The cells were scraped up in PBS, collected, centrifuged at 800 × *g* for 3 min at 4°C. The pellet containing the same amount of cell proteins was resuspended in 5 ml of ice-cold TEMP buffer (10 mM Tris-HCl, pH 7.5, 1 mM EDTA, 4 mM MgCl_2_, 0.5 mM PMSF), centrifuged again at 800 × *g* for 3 min at 4°C and resuspended in 3 ml of ice-cold TEMP buffer. After 10 min on ice, cells were dounced in tight fitting homogenizer with six to eight strokes. The nuclei were then pelleted by centrifugation at 1,500 × *g* for 10 min and the nuclear pellet was resuspended in a small volume (no more than four pellet volumes) of TEP (TEMP buffer without MgCl_2_) and an equal volume of 1 M NaCl, left on ice for 30–60 min and then spin in a cold microfuge at 13,000 × *g* for 15 min. The supernatants containing Topo II activity were assessed using kDNA provided in the TopoGEN Topo II assay kit (Topogen Inc., Port Orange, FL, United States). The assay measures the Topo II-specific enzymatic conversion of highly catenated kDNA into decatenated DNA. 1–2 μg of nuclear extracts or purified human Topo II alpha (Topo GEN), used as a positive control, were added for 30 min at 37°C to a standard reaction mixture consisting in 5X complete reaction buffer and 1 μl kDNA (250–500 ng/μl) in a final volume of 20 μl. The reaction was stopped with 4 μl stop buffer 5X. The reaction products together with linear and decatenated kDNA used as markers were resolved on a 1% w/v agarose gels for 15 min at 100V and stained with 0.01% v/v ethidium bromide. Gels were documented using Gel Doc 2000 ChemiDoc (Bio-Rad, Hercules, CA, United States) and analyzed using the Quantity One software (Bio-Rad). The appearance of a band corresponding to decatenated plasmid, that migrates rapidly into agarose gel as opposed to the circular kDNA, was taken as an index of active cellular topoisomerase II. The assay was linear between 0.25 and 1 U of purified Topo II alpha (1 U defined as the amount of enzyme that decatenates 0.2 μg of kDNA in 15 min at 37°C), and between 0.063 μg and 2 μg total protein of a reference nuclear extract.

### Statistical Analysis

Data were expressed as mean ± SEM of the mean. The results were checked for normal distribution and analyzed by one-way analysis of variance (ANOVA) followed by Tukey’s test comparing samples treated with the same dose of chemotherapeutic agent in HG vs. G condition. Statistical significance level was set at *p* = 0.05.

## Results

### Hyperglycemia Protected Against the Decreased Cell Viability and Cytotoxicity Induced by DOX and 5-FU

**FIGURE 1:**
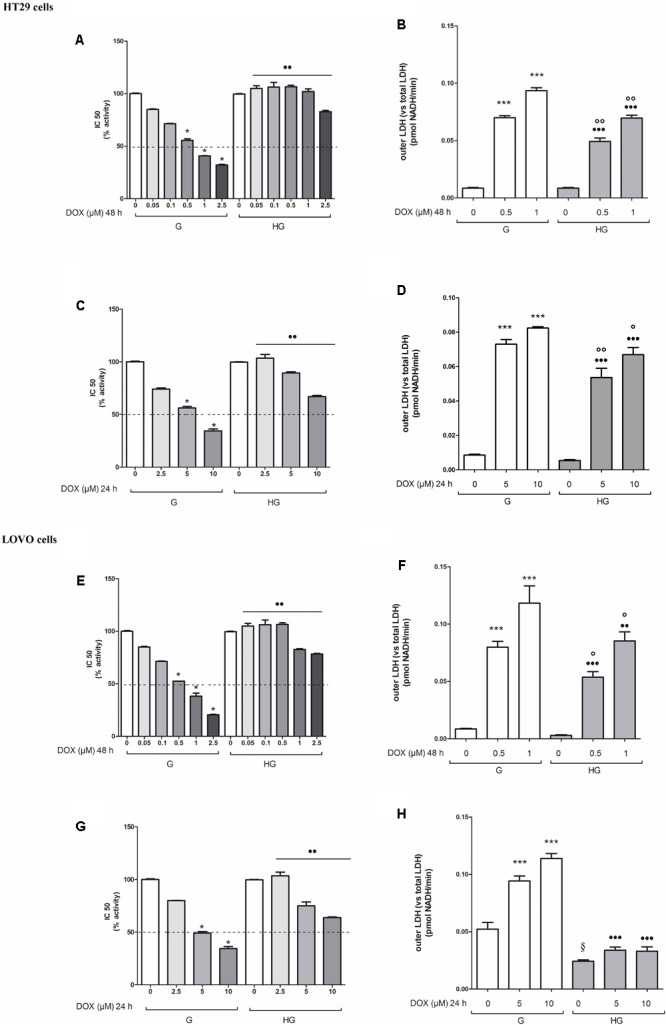
Effect of normal glucose (G) and high glucose (HG) on cell proliferation and on LDH release from cells in the supernatant in absence or presence of doxorubicin (DOX) in human colon cancer (HT29 and LOVO) cells. Cells were cultured for ≥7 days in the presence of G and HG, then subjected to the following investigations. **(A)** Cells were left untreated or incubated for 48 h in the presence of different concentrations (0.05 μM, 0.1 μM, 0.5 μM, 1 μM, 2.5 μM) of DOX **(A,E)** or 24 h in the presence of different concentrations (2.5 μM, 5 μM, 10 μM) of DOX **(C,G)**. Samples were then stained in quadruplicate with the neutral red solution (*n* = 4) to assess cell viability. IC50 was calculated as the concentration of DOX that kills 50% (dotted line) of cells. ^∗^*p* < 0.01 0.5, 1, 2.5, 5, and 10 μM DOX in HT29/LOVO cells cultured in G vs. 0 μM DOX in HT29/LOVO cells cultured in G; ^••^*p* < 0.001 0.05, 0.1, 0.5, 1, 2.5, 5, and 10 μM DOX in HT29/LOVO cells cultured in HG, respectively, vs. 0.05, 0.1, 0.5, 1, 2.5, 5, and 10 μM DOX in HT29/LOVO cells cultured in G. **(B)** Cells were left untreated or incubated for 48 h before analysis with 0.5 μM and 1 μM of DOX **(B,F)** or for 24 h with 5 μM and 10 μM of DOX **(D,H)**, then the LDH activity was measured. Extracellular LDH activity was calculated as total (intracellular and extracellular) LDH activity in the dish. Measurements (*n* = 8) were performed in duplicate. ^∗∗∗^*p* < 0.0001 0.5, 1, 5, and 10 μM DOX in HT29/LOVO cells cultured in G vs. 0 μM DOX in HT29/LOVO cells cultured in G; ^•••^*p* < 0.0001 0.5, 1, 5, and 10 μM DOX in HT29/LOVO cells cultured in HG vs. 0 μM DOX in HT29/LOVO cells cultured in HG; ∘∘*p* < 0.001 1 μM DOX in HT29/LOVO cells cultured in HG vs. 0 μM DOX in HT29/LOVO cells cultured in HG; ^∘∘^*p* < 0.001 and °*p* < 0.002, respectively, 0.5, 1, 5, and 10 μM DOX in HT29/LOVO cells cultured in HG vs. 0.5, 1, 5, and 10 μM DOX in HT29/LOVO cells cultured in G; ^§^ *p* < 0.002 0 μM DOX in LOVO cells cultured in HG vs. 0 μM DOX in LOVO cells cultured in HG.

**FIGURE 2:**
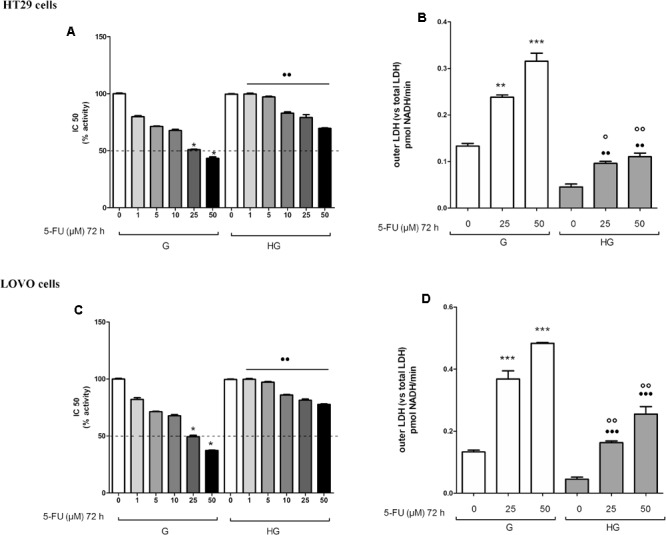
Effect of normal glucose (G) and high glucose (HG) on cell proliferation and on LDH release from cells in the supernatant in absence or presence of 5-fluorouracil (5-FU) in human colon cancer (HT29 and LOVO) cells. Cells were cultured for ≥7 days in the presence of G and HG, then subjected to the following investigations. **(A)** Cells were left untreated or incubated for 72 h in the presence of different concentrations (1 μM, 5 μM, 10 μM, 25 μM, 50 μM) of 5-FU **(A,C)**. Samples were then stained in quadruplicate with the neutral red solution (*n* = 4) to assess cell viability. IC50 was calculated as the concentration of 5-FU that kills 50% (dotted line) of cells. ^∗^*p* < 0.01 25 and 50 μM 5-FU in HT29/LOVO cells cultured in G vs. 0 μM 5-FU in HT29/LOVO cells cultured in G; ^••^*p* < 1, 5, 10, 25, 50 5-FU in HT29/LOVO cells cultured in HG, respectively, vs. 1, 5, 10, 25, 50 5-FU in HT29/LOVO cells cultured in G. **(B)** Cells were left untreated or incubated for 72 h with 25 μM and 50 μM of 5-FU **(B,D)**, then the LDH activity was measured. Measurements (*n* = 8) were performed in duplicate. ^∗∗∗^*p* < 0.0001 25 and 50 μM 5-FU in HT29/LOVO cells cultured in G vs. 0 μM 5-FU in HT29/LOVO cells cultured in G; ^∗∗^*p* < 0.001 25 μM 5-FU in HT29 cells cultured in G vs. 0 μM 5-FU in HT29 cells cultured in G; ^•••^*p* < 0.0001 25 and 50 μM 5-FU in LOVO cells cultured in HG vs. 0 μM 5-FU in LOVO cells cultured in G; ^••^*p* < 0.001 25 and 50 μM 5-FU in HT29 cells cultured in HG vs. 0 μM 5-FU in HT29 cells cultured in G; ^∘∘^*p* < 0.001 25 and 50 μM 5-FU in HT29/LOVO cells cultured in HG vs. 25 and 50 μM 5-FU in HT29 cells cultured in G; °*p* < 0.002, 25 μM 5-FU in HT29 cells cultured in HG vs. 25 μM 5-FU in HT29 cells cultured in G.

The cell viability, measured by neutral red uptake assay, was significantly higher in HT29 and LOVO cells cultured in HG compared with control cells at the same doses and time incubation of DOX (**Figures 1A,C,E,G**) or of 5-FU (**Figures 2A,C**). Only in euglycemic condition, DOX at 0.5 μM for 48 h (**Figures 1A,E**) or DOX at 5 μM for 24 h (**Figures 1C,G**, respectively, in HT29 and LOVO cells) and 5-FU at 25 μM for 72 h (**Figures 2A,C**, respectively, in HT29 and LOVO cells) were able to inhibit cell viability by 50%. Moreover, the extracellular release of the intracellular enzyme LDH, used as an index of cytotoxicity, was significantly lower in HT29 and LOVO cells cultured in HG compared with control cells at the same doses and time incubation of DOX (**Figures 1B,D,F,H**) or of 5-FU (**Figures 2B,D**), whereas, in our experimental conditions, hyperglycemia alone did not shown any cytotoxic effect. Given the superimposable data of cell viability and LDH leakage, the experimental conditions were standardized using DOX at 5 and 10 μM for 24 h and 5-FU at 25 and 50 μM for 72 h.

**FIGURE 3:**
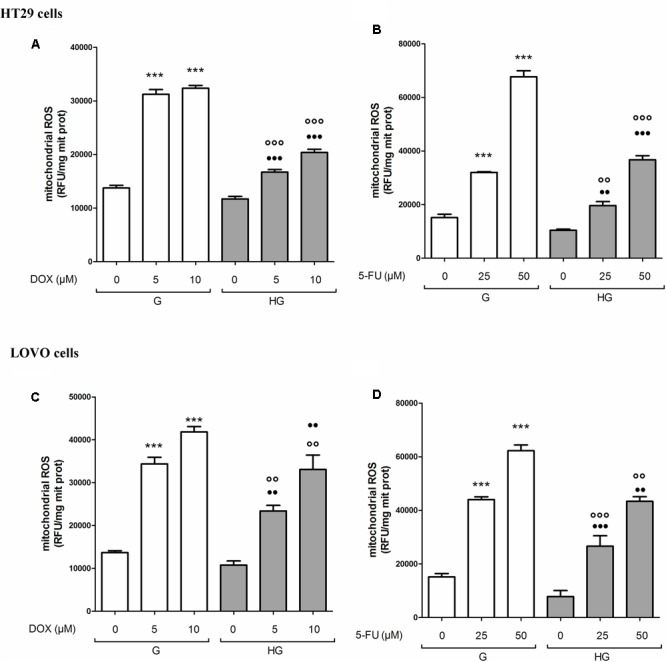
Effect of normal glucose (G) and high glucose (HG) on mitochondrial ROS production in absence or presence of DOX **(A,C)** or 5-FU **(B,D)** in human colon cancer (HT29 and LOVO) cells. Cells were cultured for ≥7 days in the presence of G and HG, incubated for 24 h before analysis with 5 and 10 μM DOX or 25 and 50 μM 5-FU and then the mitochondrial ROS levels were measured fluorimetrically in triplicate using the DCFDA-AM probe. Measurements (*n* = 8) were performed in duplicate. **(A)** ^∗∗∗^*p* < 0.0001 5 and 10 μM DOX in HT29 cells cultured in G vs. 0 μM DOX in HT29 cells cultured in G; ^∘∘∘^*p* < 0.0001 5 and 10 μM DOX in HT29 cells cultured in HG vs. 5 and 10 μM DOX in HT29 cells cultured in G; ^•••^*p* < 0.0001 5 and 10 μM DOX in HT29 cells cultured in HG vs. 0 μM DOX in HT29 cells cultured in HG. **(B)** ^∗∗∗^*p* < 0.0001 25 and 50 μM 5-FU in HT29 cells cultured in G vs. 0 μM 5-FU in HT29 cells cultured in G; ^∘∘^*p* < 0.001 25 and ^∘∘∘^*p* < 0.0001 50 μM 5-FU in HT29 cells cultured in HG vs. 25 and 50 μM 5-FU in HT29 cells cultured in G; ^••^*p* < 0.001 25 and ^•••^*p* < 0.0001 50 μM 5-FU in HT29 cells cultured in HG vs. 0 μM 5-FU in HT29 cells cultured in HG. **(C)** ^∗∗∗^*p* < 0.0001 5 and 10 μM DOX in LOVO cells cultured in G vs. 0 μM DOX in HT29 cells cultured in G; ^∘∘^*p* < 0.001 5 and 10 μM DOX in HT29 cells cultured in HG vs. 5 and 10 μM DOX in LOVO cells cultured in G; ^••^*p* < 0.001 5 and 10 μM DOX in LOVO cells cultured in HG vs. 0 μM DOX in HT29 cells cultured in HG. **(D)** ^∗∗∗^*p* < 0.0001 25 and 50 μM 5-FU in LOVO cells cultured in G vs. 0 μM 5-FU in LOVO cells cultured in G; ^∘∘∘^*p* < 0.0001 25 and ^∘∘^*p* < 0.001 50 μM 5-FU in LOVO cells cultured in HG vs. 25 and 50 μM 5-FU in LOVO cells cultured in G; ^•••^*p* < 0.0001 25 and ^••^*p* < 0.001 50 μM 5-FU in LOVO cells cultured in HG vs. 0 μM 5-FU in LOVO cells cultured in HG.

As anticancer drugs, DOX (Carlisi et al., 2017) and 5-FU (Hu et al., 2016) were shown to have important cytotoxic effects on tumor cells through ROS production, we analyzed the levels of mitochondrial ROS, the main source of cellular ROS. We found the ROS levels to be significantly lower after bolus of 5 and 10 μM DOX for 24 h and 25 and 50 μM 5-FU for 72 h in HT29 (**Figures 3A,B**) and LOVO cells (**Figures 3C,D**) cultured in HG compared with control cells. No change in total cellular ROS were found (data not shown).

### Hyperglycemia Preserved the Integrity of Mitochondrial DNA Induced by DOX and 5-FU in HT29 and LOVO Cells

**FIGURE 4:**
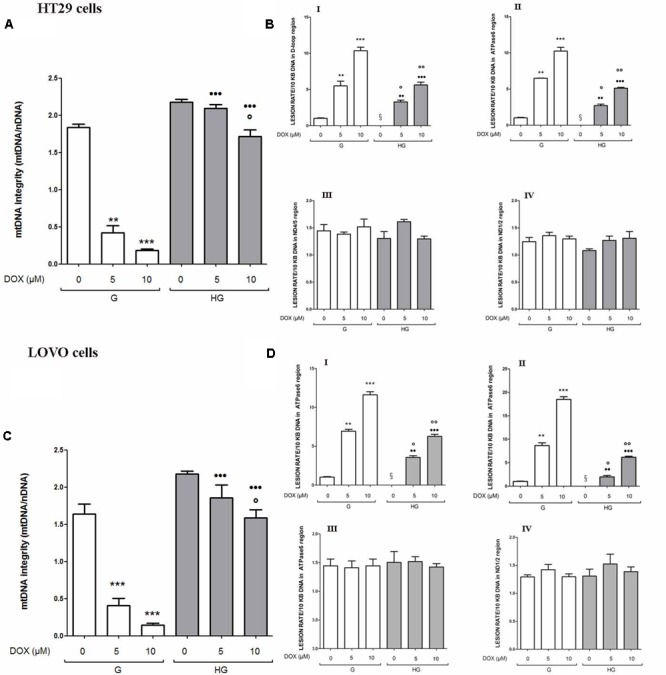
Effect of normal glucose (G) and high glucose (HG) on mitochondrial DNA damage (mtDNA) in absence or presence of DOX in human colon cancer (HT29 and LOVO) cells. Cells were cultured for ≥7 days in the presence of G and HG, incubated for 24 h before analysis with 5 and 10 μM DOX, then washed and processed to determine the mtDNA integrity by qRT-PCR and the lesion rate by semi-long run rt-PCR (SLR rt-PCR). Measurements (*n* = 6) were performed in triplicate. **(A)** For mtDNA integrity measurements: ^∗∗^*p* < 0.001 and ^∗∗∗^*p* < 0.0001, respectively, 5 and or 10 μM DOX in HT29 cells cultured in G vs. 0 μM DOX in HT29 cells cultured in G; °*p* < 0.00 10 μM DOX in HT29 cells cultured in HG vs. 0 μM DOX in HT29 cells cultured in HG; ^•••^*p* < 0.0001 5 and or 10 μM DOX in HT29 cells cultured in HG vs. 5 and or 10 μM DOX in HT29 cells cultured in G. **(C)** ^∗∗∗^*p* < 0.0001, 5 and or 10 μM DOX in LOVO cells cultured in G vs. 0 μM DOX in LOVO cells cultured in G; °*p* < 0.00 10 μM DOX in LOVO cells cultured in HG vs. 0 μM DOX in LOVO cells cultured in HG; ^•••^*p* < 0.0001 5 and or 10 μM DOX in LOVO cells cultured in HG vs. 5 and or 10 μM DOX in LOVO cells cultured in G. **(B,D)** For lesion rate measurements: ^∗∗^*p* < 0.001 and ^∗∗∗^*p* < 0.0001, respectively, 5 and or 10 μM DOX in HT29/LOVO cells cultured in G vs. 0 μM DOX in HT29/LOVO cells cultured in G; ^§^ *p* < 0.01 0 μM DOX in HT29/LOVO cells cultured in HG vs. 0 μM DOX in HT29/LOVO cells cultured in G; °*p* < 0.01 and ^∘∘^*p* < 0.001, respectively, 5 and 10 μM DOX in HT29/LOVO cells cultured in HG vs. 0 μM DOX in HT29/LOVO cells cultured in HG; ^••^ and ^•••^*p* < 0.0001, respectively, 5 and or 10 μM DOX in HT29/LOVO cells cultured in HG vs. 5 and or 10 μM DOX in HT29/LOVO cells cultured in G.

The integrity maintaining of the mitochondrial genome (mtDNA) is a prerequisite for proper mitochondrial function (Yakes and Van Houten, 1997). Due to the high concentration of mitochondrial ROS production induced by DOX or 5-FU as shown in **Figure 3**, mtDNA could be strongly exposed to oxidative stress, leading to mitochondrial DNA lesions. Therefore, mtDNA damage analysis was performed to investigate if a hyperglycemic condition, by lowering the levels of ROS induced by DOX and 5-FU, could also prevent the effect of the chemotherapeutic agents on mtDNA genome. We evaluated both the mtDNA integrity and the lesion rate in the four following different regions: (a) ATP-ase; (b) D-loop, (c) ND1/2, and (d) ND4/5. (a) and (b) are well known sites of oxidative stress damage (Rothfuss et al., 2010). We observed that in euglycemia DOX 5 and 10 μM 24 h significantly decreased the integrity of the mtDNA in a dose dependent manner, whereas in hyperglycemia only DOX 10 μM slightly decreased the integrity in HT29 and LOVO cells (**Figures 4A,C**). Furthermore, in euglycemia DOX 5 and 10 μM 24 h significantly increased the lesion rate in D-loop and encoding ATPase synthesis regions (5- and 10-fold increase, respectively), whereas in hyperglycemia the increase was only about 2.5 and 5 times, respectively (**Figures 4B,D** - I,II). Superimposable data were obtained when HT29 and LOVO cells were treated with 5-FU (data not shown). Once again, in our experimental conditions, hyperglycemia reduces the adverse effects of DOX and 5-FU by preserving the integrity of mitochondrial damage.

### Hyperglycemia Modified the MRP-1 Expression and Activity in LOVO Cells, but Not in HT29 Cells

**FIGURE 5:**
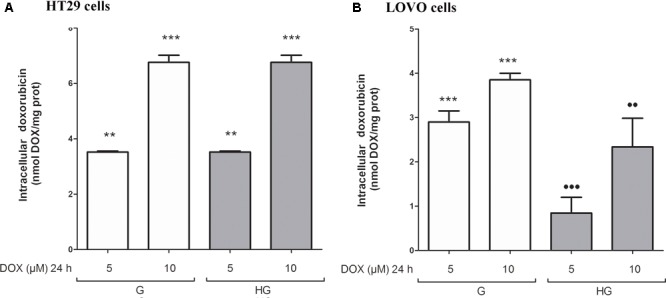
Effect of normal glucose (G) and high glucose (HG) on intracellular accumulation of DOX in human colon cancer (HT29 and LOVO) cells. Cells were cultured for ≥7 days in the presence of G and HG and incubated with 5 and 10 μM DOX for 24 h before analysis **(A,B)**. The measurements were performed in triplicate (*n* = 6). ^∗∗^*p* < 0.001 5 μM DOX in HT29 cells cultured in G and in HG vs. 0 μM DOX in HT29 cells cultured in G and in HG; ^∗∗∗^*p* < 0.0001 10 μM DOX in HT29 cells cultured in G and in HG vs. 0 μM DOX in HT29/LOVO cells cultured in G and in HG; ^∗∗∗^*p* < 0.0001 5 and 10 μM DOX in HT29 cells cultured in G vs. 0 μM DOX in HT29 cells cultured in G; ^•••^*p* < 0.0001 5 DOX in LOVO cells cultured in HG vs. 5 μM DOX in LOVO cells cultured in G; ^••^*p* < 0.001 10 DOX in LOVO cells cultured in HG vs. 10 μM DOX in LOVO cells cultured in G.

We then analyzed the effect of hyperglycemic condition on the expression and activity of the ABC transporters. In HT29 cells cultured in G or HG the amount of intracellular DOX (**Figure 5A**), analyzed spectrophotometrically, and the nuclear localization of DOX (data not shown), observed by fluorescent microscopy, were similar. These data were confirmed by HPLC analysis of cellular extracts that did not detect differences both in the intracellular content of DOX and its metabolite doxorubicinol (data not shown). At the opposite in LOVO cells, the amount of intracellular DOX (**Figure 5B**) was significantly decreased after 24 h incubation with 5 and 10 μM DOX in HG condition.

**FIGURE 6:**
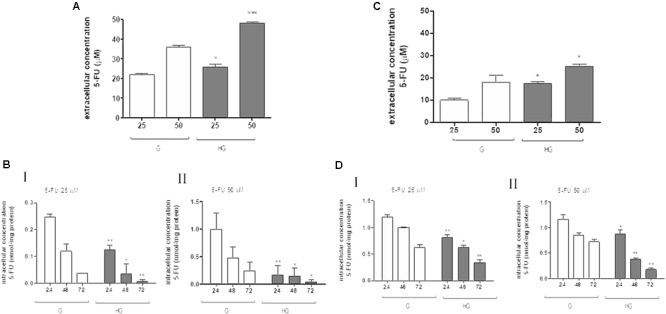
Effect of normal glucose (G) and high glucose (HG) on accumulation of 5-FU in human colon cancer HT29 and LOVO cells. Cells were cultured for ≥7 days in the presence of G and HG and incubated with 25 and 50 μM 5-FU for 72 h before analysis, then subjected to the following investigations. **(A)** The extracellular content of 5-FU **(A,C)** was assessed by HPLC. The measurements were performed in duplicate and data are presented as mean ± SEM (*n* = 4). ^∗^*p* < 0.05 25 μM 5-FU in HT29 cells cultured in HG vs. G; ^∗∗∗^*p* < 0.0001 50 μM 5-FU in HT29 cells cultured in HG vs. G. ^∗^*p* < 0.05 25 μM 5-FU in LOVO cells cultured in HG vs. G; ^∗^*p* < 0.05 50 μM 5-FU in LOVO cells cultured in HG vs. G. **(B)** The intracellular drug content of 5-FU **(B,D)** was assessed by HPLC-MS. The measurements were performed in triplicate and data are presented as mean ± SEM (*n* = 3). At 25 μM 5-FU **(B - I)**: ^∗∗^*p* < 0.001 in HT29 cells cultured in HG vs. G at 24 h; ^∗^*p* < 0.05 in HT29 cells cultured in HG vs. G at 48 h; ^∗∗^*p* < 0.001 in HT29 cells cultured in HG vs. G at 72 h. At 25 μM 5-FU **(D - I)**: ^∗∗^*p* < 0.001 in LOVO cells cultured in HG vs. G at 24 h; ^∗^*p* < 0.05 in LOVO cells cultured in HG vs. G at 48 h; ^∗∗^*p* < 0.001 in LOVO cells cultured in HG vs. G at 72 h. At 50 μM 5-FU **(B - II)**: ^∗∗^*p* < 0.001 in HT29 cells cultured in HG vs. G at 24 h; ^∗^*p* < 0.05 in HT29 cells cultured in HG vs. G at 48 h; ^∗^*p* < 0.05 in HT29 cells cultured in HG vs. G at 72 h. At 50 μM 5-FU **(D - II)**: ^∗^*p* < 0.05 in LOVO cells cultured in HG vs. G at 24 h; ^∗∗^*p* < 0.001 in LOVO cells cultured in HG vs. G at 48 h; ^∗∗^*p* < 0.001 in LOVO cells cultured in HG vs. G at 72 h.

Moreover, in HT29 and LOVO cells after 72 h of incubation with 25 and 50 μM 5-FU, the extracellular levels of 5-FU were significantly higher in the supernatants of both HT29 and LOVO cells cultured in HG condition: in HT29 cells, for 25 μM dose: 21.9 ± 1.07 in G vs. 25.9 ± 2.6 in HG and for 50 μM dose: 36.1 ± 1.3 in G vs. 48.1 ± 1.2) (**Figure 6A**), and in LOVO cells, for 25 μM dose: 10.0 ± 0.8 in G vs. 17.4 ± 0.9 in HG and for 50 μM dose: 19.6 ± 0.6 in G vs. 25.6 ± 0.7) (**Figure 6C**). The intracellular levels of 5-FU decreased in a time-dependent manner both in G and HG conditions, but the levels were lower in HG condition both in HT29 and LOVO cells (**Figures 6B,D** - I,II). Furthermore, the intracellular levels of FdUMP, one of its active metabolite, were similar in G and HG conditions (0.6 and 1 nmol/mg protein, respectively).

**FIGURE 7:**
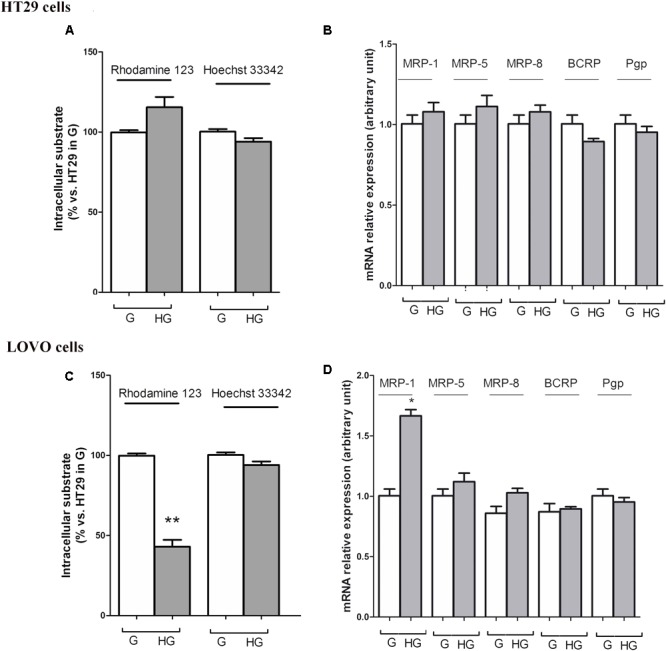
ATP binding cassette transporters activity **(A,C)** and levels of messenger RNA (mRNA) **(B,D)** of MDR-related proteins (such as BCRP and MRP1, 5 and 8) and P-glycoprotein (Pgp) genes in normal glucose (G) and high glucose (HG) in human colon cancer (HT29 and LOVO) cells. **(A,C)** Cells were cultured for ≥7 days in the presence of G and HG, then washed and maintained for further 20 min at 37°C in fresh medium or DBPS buffer, respectively, containing rhodamine 123 (to assess Pgp and MRP activity) or Hoechst 33342 (to assess BCRP activity). The cells were lysed and the intracellular fluorescence, inversely related to its efflux, was assessed fluorimetrically. Measurements (*n* = 6) were performed in triplicate. ^∗∗^*p* < 0.001 LOVO cells cultured in HG vs. LOVO cells cultured in G. **(B,D)** At the same experimental conditions cells were analyzed by quantitative real-time polymerase chain reaction (RT-qPCR). Measurements (*n* = 6) were performed in triplicate, and data, expressed as relative expression, are presented as means ± SEM; ^∗^*p* < 0.01 LOVO cells cultured in HG vs. LOVO cells cultured in G.

To confirm that the protective effect of hyperglycemia is independent of the intracellular amount of the chemotherapeutics, we observed that in HT29 cells the amount of intracellular of rhodamine 123, taken as an index of Pgp plus MRP activity, and Hoechst 33342, taken as an index of BCRP activity (**Figure 7A**), and MRP1, 5 and 8, BCRP and Pgp gene expression (**Figure 7B**) were not modified by HG incubation. Otherwise, in LOVO cells only the amount of rhodamine 123 was significantly decreased (**Figure 7C**) as well as the MRP1 gene expression were increased (**Figure 7D**).

These data showed that very HG alone, in our experimental conditions, changed neither the intracellular accumulation of DOX nor the expression of DOX and 5-FU-effluxing membrane pumps in HT29 cells, but it decreased the amount of intracellular DOX and 5-FU by increasing the activity of MRP1 through the up regulation of MRP1 expression in LOVO cells.

According to these results, HG seemed to promote drug chemoresistance in both HT29 and LOVO cells, but in a different way. If the increased expression and activity of MRP1 in LOVO cells cultured in HG could partially explain the decreased intracellular amount of DOX, the effectiveness of DOX on HT29 cells cultured in HG is to be attributed to other molecular mechanisms, among them changes in expression/function of pro- and anti-apoptotic factors, defects in the cell cycle, and/or in expression/function of the molecular targets of anticancer drugs, and enhanced ability of cancer cells to repair anticancer drug-induced DNA damage (Meyerhardt et al., 2012).

### Hyperglycemia Prevented the Susceptibility to Apoptosis and Diminished the Level of Cytosolic cyt c and the Cleavage of Full Length of PARP Induced by DOX and 5-FU in HT29 and LOVO Cells

**FIGURE 8:**
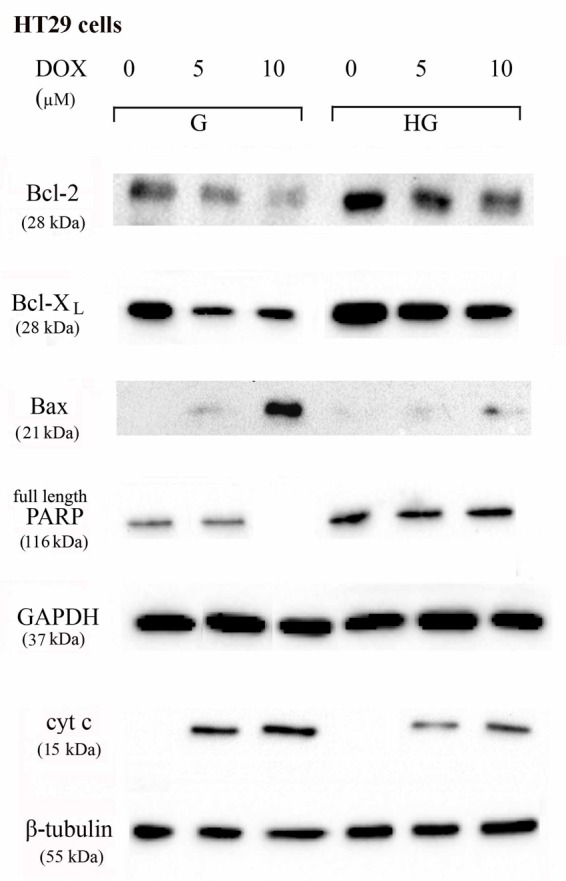
Effect of normal glucose (G) and high glucose (HG) on Bcl-2, Bcl-XL, Bax, PARP and cyt c protein expression in absence or presence of DOX in human colon cancer HT29 cells. Cells were cultured for ≥7 days in the presence of G and HG, incubated for 24 h before analysis with 5 and 10 μM DOX, then washed and lysed. The level of GAPDH, used as an housekeeping protein in total lysates, and the level of β-tubulin, used as an housekeeping protein in mitochondrial lysates, were used to check the equal protein loading. The figure is representative of three independent experiments.

**FIGURE 9:**
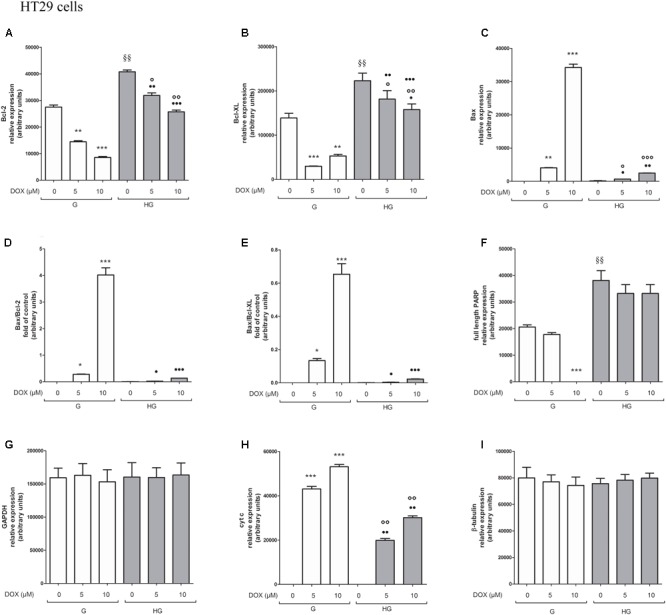
Densitometry of Bcl-2, Bcl-XL, Bax, PARP, and cyt c protein expression in absence or presence of DOX in human colon cancer HT29 cells in G and HG conditions. The protein bands of three independent experiments have been quantified by densitometry and the values are expressed as arbitrary units. **(A)** For Bcl-2 measurements: ^∗∗^*p* < 0.001 and ^∗∗∗^*p* < 0.0001 5 and 10 μM, respectively, DOX in HT29 cells cultured in G vs. 0 μM DOX in HT29 cells cultured in G; ^§§^ *p* < 0.001 0 μM DOX in HT29 cells cultured in HG vs. 0 μM DOX in HT29 cells cultured in G; °*p* < 0.001 and ^∘∘^*p* < 0.0001 5 and 10 μM DOX in HT29 cells cultured in HG vs. 0 μM DOX in HT29 cells cultured in HG; ^••^*p* < 0.001 and ^•••^*p* < 0.0001 5 and 10 μM DOX, respectively, in HT29 cells cultured in HG vs. 5 and 10 μM DOX, respectively, DOX in HT29 cells cultured in G. **(B)** For Bcl-XL measurements: ^∗∗∗^*p* < 0.0001 and ^∗∗^*p* < 0.001 5 and 10 μM, respectively, DOX in HT29 cells cultured in G vs. 0 μM DOX in HT29 cells cultured in G; ^§§^*p* < 0.001 0 μM DOX in HT29 cells cultured in HG vs. 0 μM DOX in HT29 cells cultured in G; °*p* < 0.001 and ^∘∘^*p* < 0.0001 5 and 10 μM DOX in HT29 cells cultured in HG vs. 0 μM DOX in HT29 cells cultured in HG; ?*p* < 0.01 10 μM DOX in HT29 cells cultured in HG vs. 10 μM DOX in HT29 cells cultured in G. **(C)** For Bax measurements: ^∗∗∗^*p* < 0.0001 and ^∗∗^*p* < 0.001 5 and 10 μM, respectively, DOX in HT29 cells cultured in G vs. 0 μM DOX in HT29 cells cultured in G; °*p* < 0.001 and ^∘∘∘^*p* < 0.0001 5 and 10 μM DOX in HT29 cells cultured in HG vs. 0 μM DOX in HT29 cells cultured in HG; ?*p* < 0.001 and ^••^*p* < 0.001 and ^•••^*p* < 0.0001 5 and 10 μM DOX, respectively, in HT29 cells cultured in HG vs. 5 and 10 μM DOX, respectively, DOX in HT29 cells cultured in G. **(D)** For Bax/Bcl-2 ratio measurements: ^∗^*p* < 0.001 and ^∗∗∗^*p* < 0.0001 5 and 10 μM, respectively, DOX in HT29 cells cultured in G vs. 0 μM DOX in HT29 cells cultured in G; ?*p* < 0.001 and ^•••^*p* < 0.0001 5 and 10 μM, respectively, DOX in HT29 cells cultured in HG vs. 5 and 10 μM DOX in HT29 cells cultured in G. **(E)** For Bax/Bcl-XL ratio measurements: ^∗^*p* < 0.01 and ^∗∗∗^*p* < 0.0001, respectively, 5 and or 10 μM DOX in HT29 cells cultured in G vs. 0 μM DOX in HT29 cells cultured in G; ?*p* < 0.001 and ^•••^*p* < 0.0001, respectively, 5 and or 10 μM DOX in HT29 cells cultured in HG vs. 5 and or 10 μM DOX in HT29 cells cultured in G. **(F)** For full length PARP measurements: ^∗∗∗^*p* < 0.0001 10 μM DOX in HT29 cells cultured in G vs. 0 μM DOX in HT29 cells cultured in G; ^§§^ *p* < 0.001 0 μM DOX in HT29 cells cultured in HG vs. 0 μM DOX in HT29 cells cultured in G. **(H)** For cit cyt c measurements: ^∗∗∗^*p* < 0.0001 5 and 10 μM, respectively, DOX in HT29 cells cultured in G vs. 0 μM DOX in HT29 cells cultured in G; ^∘∘^*p* < 0.001 5 and 10 μM DOX in HT29 cells cultured in HG vs. 0 μM DOX in HT29 cells cultured in HG; ^••^*p* < 0.0001 5 and 10 μM DOX, respectively, in HT29 cells cultured in HG vs. 5 and 10 μM DOX, respectively, DOX in HT29 cells cultured in G. **(G,I)** For GAPDH and β-tubulin measurements: n.s.

**FIGURE 10:**
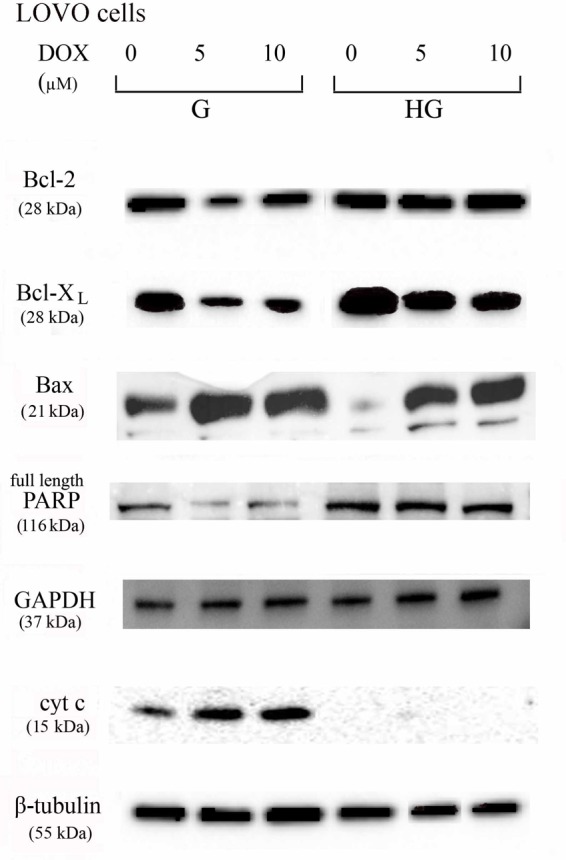
Effect of normal glucose (G) and high glucose (HG) on Bcl-2, Bcl-XL, Bax, PARP and cyt c protein expression in absence or presence of DOX in human colon cancer LOVO cells. Cells were cultured for ≥7 days in the presence of G and HG, incubated for 24 h before analysis with 5 and 10 μM DOX, then washed and lysed. The level of GAPDH, used as an housekeeping protein in total lysates, and the level of β-tubulin, used as an housekeeping protein in mitochondrial lysates, were used to check the equal protein loading. The figure is representative of three independent experiments.

**FIGURE 11:**
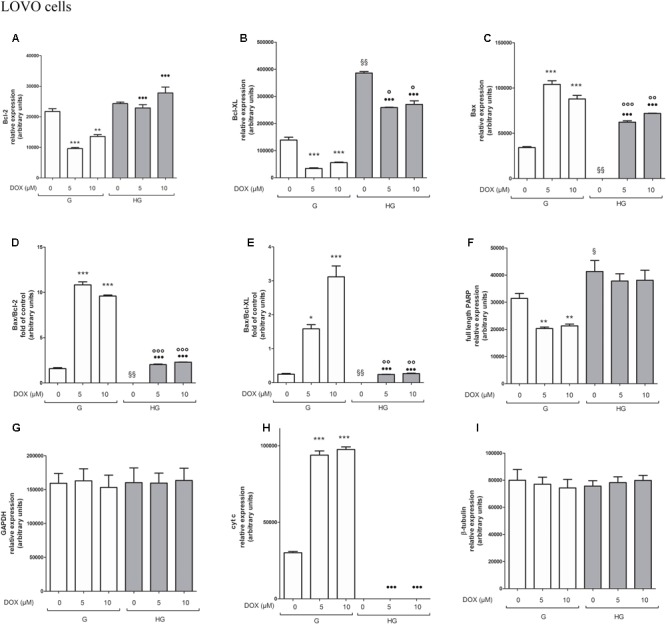
Densitometry of Bcl-2, Bcl-XL, Bax, PARP and cyt c protein expression in absence or presence of DOX in human colon cancer LOVO cells in G and HG conditions. The protein bands of three independent experiments have been quantified by densitometry and the values are expressed as arbitrary units. **(A)** For Bcl-2 measurements: ^∗∗∗^*p* < 0.0001 and ^∗∗^*p* < 0.001 5 and 10 μM, respectively, DOX in HT29 cells cultured in G vs. 0 μM DOX in HT29 cells cultured in G; ^•••^*p* < 0.0001 5 and 10 μM DOX, respectively, in HT29 cells cultured in HG vs. 5 and 10 μM DOX, respectively, DOX in HT29 cells cultured in G. **(B)** For Bcl-XL measurements: ^∗∗∗^*p* < 0.0001 5 and 10 μM, respectively, DOX in HT29 cells cultured in G vs. 0 μM DOX in HT29 cells cultured in G; ^§§^ *p* < 0.001 0 μM DOX in HT29 cells cultured in HG vs. 0 μM DOX in HT29 cells cultured in G; °*p* < 0.001 5 and 10 μM DOX in HT29 cells cultured in HG vs. 0 μM DOX in HT29 cells cultured in HG; ^•••^*p* < 0.0001 5 and 10 μM DOX in HT29 cells cultured in HG vs. 5 and 10 μM DOX in HT29 cells cultured in G. **(C)** For Bax measurements: ^∗∗∗^*p* < 0.0001 5 and 10 μM, respectively, DOX in HT29 cells cultured in G vs. 0 μM DOX in HT29 cells cultured in G; ^§§^ *p* < 0.001 0 μM DOX in HT29 cells cultured in HG vs. 0 μM DOX in HT29 cells cultured in G; ^∘∘∘^*p* < 0.0001 and ^∘∘^*p* < 0.001 5 and 10 μM DOX in HT29 cells cultured in HG vs. 0 μM DOX in HT29 cells cultured in HG; ^•••^*p* < 0.0001 5 and 10 μM DOX, respectively, in HT29 cells cultured in HG vs. 5 and 10 μM DOX, respectively, DOX in HT29 cells cultured in G. **(D)** For Bax/Bcl-2 ratio measurements: ^∗∗∗^*p* < 0.0001 5 and 10 μM, respectively, DOX in HT29 cells cultured in G vs. 0 μM DOX in HT29 cells cultured in G; ^§§^ *p* < 0.001 0 μM DOX in HT29 cells cultured in HG vs. 0 μM DOX in HT29 cells cultured in G; ^∘∘∘^*p* < 0.001 5 and 10 μM DOX in HT29 cells cultured in HG vs. 0 μM DOX in HT29 cells cultured in HG; ^•••^*p* < 0.0001 5 and 10 μM, respectively, DOX in HT29 cells cultured in HG vs. 5 and 10 μM DOX in HT29 cells cultured in G. **(E)** For Bax/Bcl-XL ratio measurements: ^∗^*p* < 0.01 and ^∗∗∗^*p* < 0.0001, respectively, 5 and or 10 μM DOX in HT29 cells cultured in G vs. 0 μM DOX in HT29 cells cultured in G; ^§§^ *p* < 0.001 0 μM DOX in HT29 cells cultured in HG vs. 0 μM DOX in HT29 cells cultured in G; ^∘∘^*p* < 0.001 5 and 10 μM DOX in HT29 cells cultured in HG vs. 0 μM DOX in HT29 cells cultured in HG; ^•••^*p* < 0.0001, respectively, 5 and or 10 μM DOX in HT29 cells cultured in HG vs. 5 and or 10 μM DOX in HT29 cells cultured in G. **(F)** For full length PARP measurements: ^∗∗^*p* < 0.001 5 and 10 μM, respectively, DOX in HT29 cells cultured in G vs. 0 μM DOX in HT29 cells cultured in G; ^§^ *p* <0.01 0 μM DOX in HT29 cells cultured in HG vs. 0 μM DOX in HT29 cells cultured in G. **(H)** For cit cyt c measurements: ^∗∗∗^*p* < 0.0001 5 and 10 μM, respectively, DOX in HT29 cells cultured in G vs. 0 μM DOX in HT29 cells cultured in G; ^•••^*p* < 0.0001 5 and 10 μM DOX, respectively, in HT29 cells cultured in HG vs. 5 and 10 μM DOX, respectively, DOX in HT29 cells cultured in G. **(G,I)** For GAPDH and β-tubulin measurements: n.s.

To verify the effect of hyperglycemia on ROS-mediated mitochondrial pathway, we focused our attention on the expression of pro- (Bax) and anti-apoptotic (Bcl-2 and Bcl-XL) proteins that are involved not only in permeability of the outer mitochondrial membrane, but also, through their effectors, in the regulation of the cell cycle, DNA repair and replication (Donovan and Cotter, 2004). The basal levels of Bcl-2 and Bcl-XL were higher in HT29 (**Figures 8, 9** and **Supplementary Figures S1, S2**) and LOVO cells (**Figures 10, 11** and **Supplementary Figures S3, S4**) cultured in HG than that of cells cultured in normal glucose. The addition of DOX at 5 and 10 μM for 24 h and of 5-FU at 50 μM for 72 h down regulated much more the expression of Bcl-2 and Bcl-XL in conditions of euglycemia than in hyperglycemia in both HT29 and LOVO cells. Moreover, the expression of Bax was completely absent in HT29 cells cultured in HG in presence of 5 μM DOX and very low in presence of 10 μM DOX (**Figures 8, 9**). Even if LOVO cells showed a higher basal level of Bax, the addition of DOX at 5 and 10 μM for 24 h upregulated the expression of Bax in conditions of euglycemia and hyperglycemia (**Figures 10, 11**). In HT29 cells after 50 μM 5-FU incubation Bax expression was significantly increased in G condition and very low in HG condition (**Supplementary Figures S1, S2**), whereas in LOVO cells this increase is abolished (**Supplementary Figures S3, S4**). Considering the increase of the ratio Bax/Bcl-2 and Bax/Bcl-XL as an indicator of susceptibility to apoptosis, in both HT29 and LOVO cells, the state of hyperglycemia abolished the pro-apoptotic effects of DOX and 5-FU by decreasing the ratio Bax/Bcl-2 and Bax/Bcl-XL (**Figures 9, 11** and **Supplementary Figures S2, S4**).

Furthermore, there was a significant decrease in the release of cytochrome c (cyt c) in the cytosol after bolus of DOX 5 and 10 μM and 5-FU 25 and 50 μM in HT29 and LOVO cells cultured in HG. This data is in agreement with the decreased expression of Bax, known mediator of the release of some factors, including the cyt c, able to trigger apoptosis if they are located in the cytosol (**Figures 8–11** and **Supplementary Figures S1–S4**).

We next used Poly ADP-ribose polymerase (PARP) as caspase activation index and as enzyme involved in DNA repair. In hyperglycemia, we did not observe any enzymatic cleavage of full length of PARP after DOX and 5-FU incubation of HT29 and LOVO cells, whereas in euglycemia the presence of DOX for 24 h and 5-FU for 72 h lead to a decrease (5 μM DOX, 25 μM 5-FU) or disappearance (10 μM DOX, 50 μM 5-FU) of the full length PARP in HT29 cells (**Figures 8, 9** and **Supplementary Figures S1, S2**) and in LOVO cells (**Figures 10, 11** and **Supplementary Figures S3, S4**).

### Hyperglycemia Reduced Cell Death by Decreasing the Percentage of Cells in Sub-G1 Peak Induced by DOX and 5-FU in HT29 and LOVO Cells

As these anti-apoptotic factors are also involved in the regulation of the cell cycle, we then performed experiments to evaluate cell cycle in euglycemic and hyperglycemic conditions.

Cell cycle arrest in the G2/M phase, decrease of G0/G1 population and subsequent increase of cell percentage in sub-G1 induced by DOX was reverted by hyperglycemia

**FIGURE 12:**
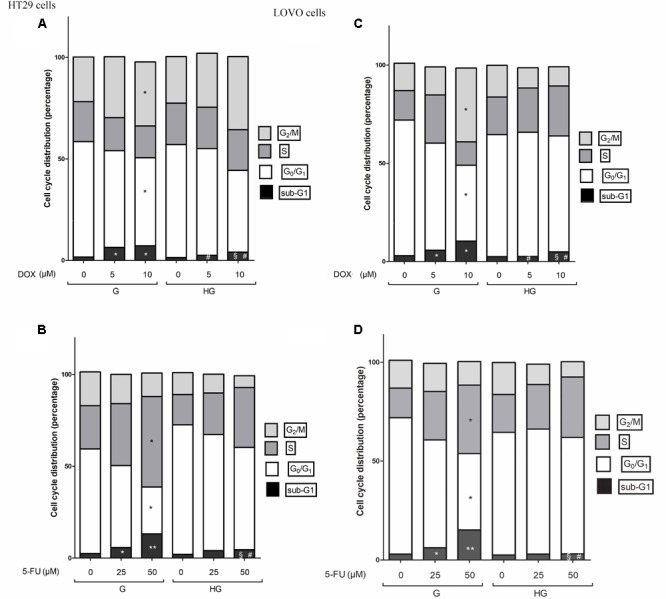
Effect of normal glucose (G) and high glucose (HG) on cell cycle in HT29 and LOVO cells in absence or presence of DOX **(A,C)** or 5-FU **(B,D)**. Cells were cultured for ≥7 days in the presence of G and HG, incubated for 24 h before analysis with 5 and 10 μM DOX **(A,C)** or for 72 h with 25 and 50 μM 5-FU **(B,D)** and then cells were analyzed for DNA content by FACS analysis. Panels represent the distribution of cells in different phases of cell cycle. Measurements (*n* = 3) were performed and data are presented as means ± SEM. **(A,C)** ^∗^*p* < 0.01 5 and 10 μM DOX in HT29 cells cultured in G vs. 0 μM DOX in HT29 cells cultured in G; ^§^ *p* < 0.01 10 μM DOX in HT29 cells cultured in HG vs. 0 μM DOX in HT29 cells cultured in HG; ^#^*p* <0.01 5 and 10 μM DOX in HT29 cells cultured in HG vs. 5 and 10 μM DOX in HT29 cells cultured in G. **(B,D)** ^∗^*p* < 0.01 and ^∗∗^*p* < 0.001 25 and 50 μM 5-FU in HT29 cells cultured in G vs. 0 μM 5-FU in HT29 cells cultured in G; ^§^ *p* < 0.01 50 μM 5-FU in HT29 cells cultured in HG vs. 0 μM 5-FU in HT29 cells cultured in HG; ^#^*p* < 0.01 50 μM 5-FU in HT29 cells cultured in HG vs. 50 μM 5-FU in HT29 cells cultured in G.

In HT29 and LOVO cells cultured in normal glucose the 24 h DOX treatment at 10 μM dose induced a significant accumulation of the cells in the G2/M phase, demonstrating inhibition of cell proliferation by cycle arrest in this phase, accompanied by a corresponding decrease of G0/G1 population, and causing cell death as shown by an increase in the percentage of cells in sub-G1 peak (**Figures 12A,C**). At 5 and 10 μM DOX in G condition, we assisted at a significant increase of percentage of cells in sub-G1 compared to HG condition. Moreover, the DOX treatment in HG condition did not influence the percentage of cells in G0/G1, S and G2/M.

Cell cycle arrest in the S phase, decrease of G0/G1 population and subsequent increase of cell percentage in sub-G1 induced by 5-FU and its metabolites was reverted by hyperglycemia

In HT29 and LOVO cells cultured in normal glucose a 72 h treatment with 50 μM 5-FU led to a significant increase in the percentage of cells in S-phase suggesting a strong cell cycle arrest in this phase and a corresponding significant decrease of G0/G1 population, accompanied by a significant increase of sub-G1 cell death population (**Figures 12B,D**). Moreover, this latter effect was visible also in cells incubated with 25 μM 5-FU. On the opposite, the 5-FU treatment in HG condition did not influence the percentage of cells in G0/G1, S and G2/M and the percentage of cell death is significantly lower at 50 μM compared to G condition, even if at this concentration the percentage of cells in sub-G1 peak remained significantly increased compared to HG condition in the absence of 5-FU.

### Hyperglycemia Lowered the Expression and Activity of Topoisomerase Abrogating the Action of DOX

**FIGURE 13:**
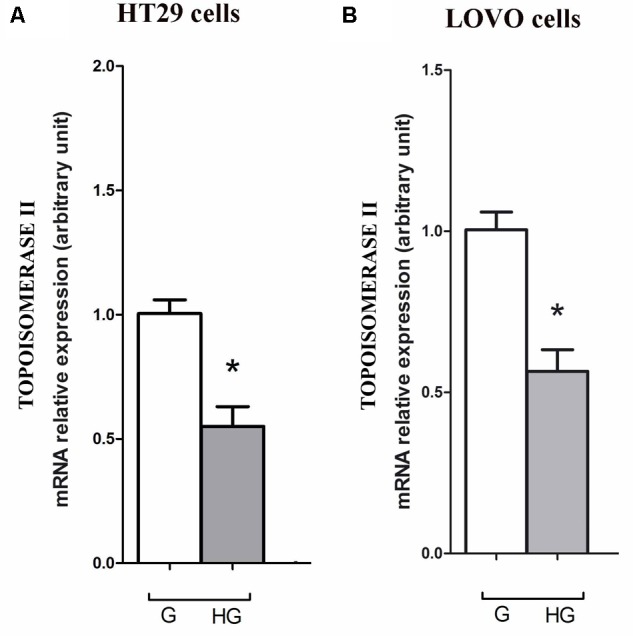
Effect of normal glucose (G) and high glucose (HG) on mRNA expression and in absence or presence of DOX in human colon cancer (HT29 **(A)** and LOVO **(B)**) cells. Cells were cultured for ≥7 days in the presence of G and HG, then washed and processed to determine the Topoisomerase II mRNA expression. The figure is representative of three independent experiments. Cells were analyzed by quantitative real-time polymerase chain reaction (RT-qPCR). Measurements (*n* = 6) were performed in triplicate, and data are expressed as relative expression; **(A)** ^∗^*p* < 0.01 HT29 cells cultured in HG vs. HT29 cells cultured in G and **(B)** ^∗^*p* < 0.01 LOVO cells cultured in HG vs. LOVO cells cultured in G.

As DOX could act as an intercalating agent of DNA synthesis blocking its transcription and inhibiting the nuclear enzyme topoisomerase activity of type II (Topo II alpha), we evaluated the effect of hyperglycemia on the Topo II alpha expression and activity. The RT-PCR results showed that hyperglycemia decreased the expression of TOPO II alpha mRNA both in HT29 and LOVO cells (**Figures 13A,B**). Moreover, we tested the activity of the topoisomerase II which, if presents in the nuclear lysate, is able to decatenate, in the presence of ATP, the kDNA (catenate DNA provided in the Topoisomerase II Assay Kit as a substrate of the reaction) in decatenated DNAs that migrate rapidly into an agarose gel.

**FIGURE 14:**
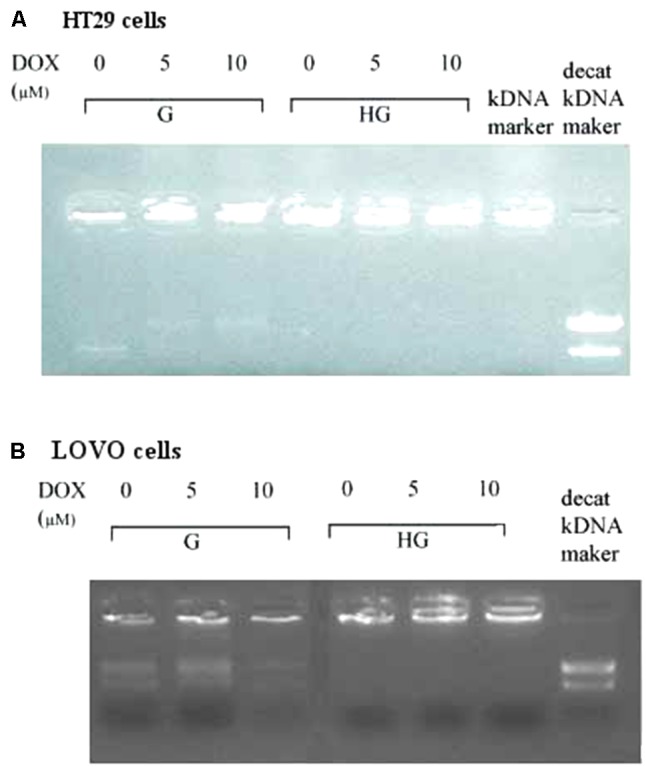
Effect of normal glucose (G) and high glucose (HG) on Topoisomerase II alpha activity in absence or presence of DOX in human colon cancer (HT29 **(A)** and LOVO **(B)**) cells. Cells were cultured for ≥7 days in the presence of G and HG, incubated for 24 h before analysis with 5 and 10 μM DOX, then washed and processed to determine the nuclear Topoisomerase II catalytic activity. The figure is representative of three independent experiments. The nuclear Topo II alpha activity is represented by the reduction of catenated kDNA provided in TopoGEN assay kit and/or the appearance of decatenated kDNA.

In HT29 and LOVO cells (**Figures 14A,B**), the activity of topoisomerase II, corresponding to a higher decatenation of kDNA, after 24 h incubation of DOX 5 and 10 μM, is completely inhibited in euglycemia. In hyperglycemia this activity was lower in absence of DOX, due to a decrease of the expression of TOPO II alpha mRNA, and 24 h incubation of DOX did not anymore affect enzymatic activity.

## Discussion

Many epidemiological data clearly indicate that the risk of several types of cancer is increased in diabetic patients and a number of cancer types have shown a higher mortality rate in patients with hyperglycemic associated pathologies (Vasconcelos-Dos-Santos et al., 2017), even if less is known about the effect of hyperglycemia on the response to the treatment of cancer.

Nowadays, some studies show that hyperglycemia confers resistance to drug-induced cell death in different cancer cells through different mechanisms. Garufi and D’ Oraz (2014) has reported that a high blood glucose condition negatively affects tumor response to therapies through the specific inhibition of p53 – Ser 46 phosphorylation thus reducing p53 apoptotic activity in colon, lung and ovarian cancer cell lines treated with chemotherapeutic agents; Zeng et al. (2010) has shown that hyperglycemia reduces the efficacy of chemotherapeutic drugs in malignant breast epithelial cells through the activation of the fatty acid synthase (FAS), enzyme responsible for the *de novo* synthesis of fatty acids from sugars and the expression of which positively correlates with aggressive tumors and poorer prognoses in various cancers (Kuhajda, 2000). Biernacka et al. (2013) has demonstrated the importance of metabolic environment in reversible epigenetic modifications to genes that affect treatment efficacy as increased glucose concentrations inhibits the efficacy of docetaxel at inducing apoptosis in prostate cancer cell lines by an increase of IGF-binding protein 2 (IGFBP2) production, as a result of glucose-induced acetylation of histones bound to the IGFBP2 promoter; Shao et al. (2014) has demonstrated that high concentrations of glucose suppress etoposide-induced cell death of B-cell lymphoma by inducing the transcription factor BCL-6 that acts as a transcriptional repressor of etoposide-induced apoptosis via the caspase pathway. Moreover, Ma et al. (2014) has determined that HG treatment diminishes the antiproliferative effect of 5-FU on colon cancer cells by decreasing cell death and increasing DNA replication, suggesting that in diabetic patients with colorectal cancer a higher dosage and duration of 5-FU treatment is required to inhibit the growth of tumor cells adequately. More recently, Ikemura and Hashida (2017) has reported that anticancer agents oxaliplatin and 5-FU administered to streptozotocin (STZ)-treated hyperglycemic mice are less effective and their survival is shorter compared to STZ-treated euglycemic mice.

ROS may play dual role in cancer progression in a dose-dependent manner. On the one hand, mild intracellular ROS can stimulate tumor progression by promoting cell proliferation, survival, invasion and metastasis; on the other hand, excess ROS production can cause oxidative damage and trigger cancer cell death (Nishikawa et al., 2009). Indeed it has been observed that HG levels increased ROS production by activating both p38 MAPK and ERK pathways (Li et al., 2014), and by stimulating cell proliferation, migration and invasion (Flores-López et al., 2016), respectively, in pancreatic and breast cancer cells. In contrast, as known, chemotherapeutic agents inducing an oxidative stress status cause mitochondrial respiratory chain ROS generation (Victorino et al., 2014) that play a significant role in the inhibition of cancer progression (Trachootham et al., 2009). Moreover, due to the high reactive environment, and probably to a mitochondrial chromatin-like structure condensed to a lesser extent than nuclear chromatin, the mtDNA is frequently exposed to oxidative stress leading to mitochondrial genomic defects and any damage within the electron transport system (Yakes and Van Houten, 1997). Thus, these alterations in mitochondrial physiology may contribute to a cellular stress response, cell growth arrest, and subsequent apoptosis (Rothfuss et al., 2010).

For the first time, we demonstrate that a prolonged HG exposure protects human colon adenocarcinoma HT29 and LOVO cells against the decreased cell viability and cytotoxicity by, at least partly, lowering mitochondrial ROS production induced by DOX and 5-FU, thus impairing the effectiveness of the chemotherapy itself. Moreover, our results provide support for the role of mitochondrial derived ROS drug-induced in mtDNA damage during G growth condition, whereas under HG exposure, the small amount of generated ROS within the mitochondria leads to a low level of mtDNA damage in the D-loop and ATPase6 regions.

In addition to this evidence, as we could not exclude that HG exposure also acted on the onset of MDR through the overexpression of ABC transporters, we investigated whether the effect of hyperglycemia on the decreased cell viability and cytotoxicity induced by DOX and 5-FU may be explained by a change in the expression and activity of Pgp and/or the MDR-related proteins, among which MRP1, 5 and 8, and BCRP. We do not observe any difference neither in the amount of intracellular content of DOX and their metabolites nor in the localization of DOX when HT29 cells are cultured in G or HG conditions. In agreement with these results, we observe that in HT29 cells the amount of intracellular of rhodamine 123, taken as an index of Pgp plus MRP activity, and Hoechst 33342, taken as an index of BCRP activity, and MRP1, 5 and 8, BCRP and Pgp gene expression are not modified by HG incubation in HT29 cells. However, in LOVO cells, we observe that HG exposure decreases the amount of intracellular DOX, increasing the activity of MRP1 through the up regulation of MRP1 expression. These latter data with the results obtained in a murine tumor model in which glucose-exposed tumor cells of late tumor-bearing stage show a declined susceptibility to the cytotoxic action of cisplatin and methotrexate, accompanied by an increased expression of MDR-1 gene (Vishvakarma et al., 2013). Moreover our results totally deviate from the study of Pandey et al. (2011) in which it has been reported that hyperglycemia potentiates the cytotoxicity of carboplatin- and 5-FU-treated breast cells cultured in HG by increasing ROS levels and reducing the expression of P-glycoprotein that promotes cell killing by increasing drug accumulation. Seebacher et al. (2015) suggested that, in small cell lung and cervical carcinoma cell lines, glucose deprivation or HG induce stress, increasing HIF-1α and leading to a more aggressive MDR phenotype via up-regulation of Pgp.

At this stage, we have no explanation on why HG induces drug chemo-resistance in a different manner in HT29 and LOVO cells. Nevertheless, if the effect of HG in LOVO cells on the response to chemotherapeutic drugs seems to follow a well-honed mechanism, the effectiveness of DOX and 5-FU on HT29 cells cultured in HG is to be attributed to other molecular mechanisms, independent from the overexpression ABC transporters, among them changes in expression/function of pro- and anti-apoptotic factors, defects in the cell cycle, and/or in expression/function of the molecular targets of anticancer drugs and enhanced ability of cancer cells to repair anticancer drug-induced DNA damage (Meyerhardt et al., 2003).

As is the case with most of the chemotherapeutic agents, multiple mechanisms have been implicated in the development of drugs resistance, including elevated levels of anti-apoptotic genes, alterations in permeability of the outer mitochondrial membrane and through their effectors in the regulation of the cell cycle (Donovan and Cotter, 2004) and an increase in DNA damage repair (Fojo and Bates, 2003). Apoptosis can be of two types, one involving engagement of the death receptors and the other with initiated and propagated by generation of ROS, and consequently the release of cytochrome c from the mitochondria (Samejima et al., 2001). The Bcl-2 protein is known to inhibit apoptosis by regulating the mitochondrial membrane potential ΔψM and cytochrome c release needed for activation of caspase-9 (Yoon et al., 2013). Panaretakis et al. (2002) reported that Bcl-2 overexpression protects against DOX-induced apoptosis through blockage of the activation of Bad and Bax.

The intracellular effects of DOX include free radical formation, activation of caspases, cleavage of Poly ADP-ribose polymerase (PARP), inhibition of DNA topoisomerase II and also nucleotide intercalation, resulting in inhibition of DNA replication, presence of DNA fragmentation and sub-diploid DNA content (Poornima et al., 2014). Recently, Zhao et al. (2015) demonstrated that, in gastric cancer cells, hyperglycemia may confer MDR at least partially by increasing the nicotinamide phosphoribosyltransferase (Nampt) and the silent information regulator 1 (Sirt1) expression, promoting Pgp level and reducing Topo IIα expression. Zhao et al. (2015) has reported that Topo II alpha mRNA expression was higher compared with gastric cancer patients. Our data demonstrated that hyperglycemia lowered the mRNA expression and activity of Topo II alpha, thus abrogating the action of DOX both in HT29 and LOVO colon adenocarcinoma cells.

We therefore focus our attention on the expression of pro- (Bax) and anti-apoptotic (Bcl-2 and Bcl-XL) proteins and, as expected, we confirm that the state of hyperglycemia abolishes the pro-apoptotic effects of DOX and 5-FU by decreasing, in both HT29 and LOVO cells, the ratio Bax/Bcl-2 and Bax/Bcl-XL, taken as an indicator of susceptibility to apoptosis. In agreement with this, the release of cytochrome c in the cytosol after bolus of DOX and 5-FU is decreased in HG condition. Moreover, the basal level PARP, used as caspase activation index and as enzyme involved in DNA repair, is significantly higher in hyperglycemia and we did not observed in this condition any enzymatic cleavage of full length of PARP after DOX and 5-FU incubation of HT29 and LOVO cells.

Kuznetsov et al. (2011) demonstrated that DOX inhibits human colon and breast cancer cell proliferation through cell cycle arrest at the G2/M phase in a time- and dose-dependent manner, also causing massive cell death. The acquisition of drug resistance of cancer cells is a major obstacle in cancer treatment also due to differences in cell cycle modulation and progression. In our study, we observed that in hyperglycemia there is a significant lower percentage of cells in sub-G1 population compared to G condition and the DOX and 5-FU treatment in HG condition did not influence the percentage of cells in G0/G1, S and G2/M, suggesting that hyperglycemia may modify the DOX or 5-FU effect modulating the cyclin–dependent cell growth checkpoint.

Three of the 5-FU intracellular formed nucleotides metabolites, the 5-fluorouridine 5-triphosphate (FUTP), the 5-fluoro-2-deoxyuridine 5-triphosphate (FdUTP), and the 5-fluoro-2-deoxyuridine 5-monophosphate (FdUMP), are responsible for the antineoplastic effect of 5-FU. In brief, FUTP is incorporated into RNA and interferes with normal RNA processing and function, FdUTP is incorporated into DNA, leading to pathological DNA structures and ultimately cell death and FdUMP acts during the S phase of the cell cycle inhibiting DNA synthesis by restricting availability of thymidylate as it inhibits the thymidylate synthetase (Derissen et al., 2015). Moreover 5-FU increases ROS production which triggered the mitochondria-caspase pathway of apoptosis (Hu et al., 2016). Matuo et al. (2009) shown that 5-FU and FdUMP influenced differently the cell cycle progression of human SW620 colon adenocarcinoma cell line. Indeed, 5-FU induced a G1/S arrest, while FdUMP led to a G2/M arrest. This different pattern of cell cycle arrest suggests that the two drugs induce different types of primary DNA lesions, leading to the activation of different checkpoints and to the recruitment of different DNA repair pathways (Matuo et al., 2009). In HT29 and LOVO cells cultured in normal glucose, only after 72 h treatment with 25 and 50 μM 5-FU, there is a significant increase in the percentage of cells in S-phase suggesting a strong cell cycle arrest in this phase and a corresponding significant decrease of G0/G1 population, accompanied by a significant increase of sub-G1 cell death population, LDH leakage and ROS production. On the opposite, in HG condition 5-FU does not influence the percentage of cells in G0/G1, S and G2/M and that of cell death is significantly lower also at 50 μM compared to G condition, accompanied by low levels of LDH leakage and ROS. Moreover, our data show that also in G condition, FdUMP does not have any effect on cell cycle arrest in G2/M, probably due to its very low level of synthesis. Furthermore, as there are no differences in the expression/activity of MRP5 and 8 between G and HG conditions, our data, confirmed by the measurement of the intracellular amount of 5-FU, suggest that the lack of 5-FU treatment effect in HG condition is probably due to the lower entry of the drug into the cells.

Taken together, these data bring further knowledge on the molecular mechanisms involved in chemoresistance during hyperglycemia. Indeed a prolonged exposure to HG protects human colon adenocarcinoma cells from the cytotoxic effects of two widely used chemotherapeutic drugs, impairing the effectiveness of the chemotherapy itself.

This should elicit an even greater care in maintaining the euglycemic state in pre- and diabetic oncological patients to increase the success of chemotherapy, thus limiting the over dose of the treatment with the subsequent side effects.

## Author Contributions

LB performed the following experiments: measurement of total cellular and mitochondrial ROS production, mitochondrial DNA (mtDNA) damage, topoisomerase II alpha assay, real-time polymerase chain reaction, and western blot experiments. She contributed to the statistical analysis, the interpretation of the results, and to write the manuscript. EM performed the following experiments: cell viability, lactate dehydrogenase (LDH) leakage and DOX accumulation and Pgp, MRP and BCRP activities and western blot experiments. She participated in the discussion of the results and contributed to draft the manuscript. RM performed the following experiments: cell viability, lactate dehydrogenase (LDH) leakage and DOX accumulation. OB performed preparation of samples and the measurements of cell cycle. She discussed the results concerning cell cycle analysis. BR performed preparation of samples and the measurements of RP-HPLC quantification of DOX and 5-FU and their metabolites. She discussed the results concerning RP-HPLC analysis. SD conceived the study, performed immunofluorescence staining experiments, contributed to the final interpretation of the data and to write the manuscript. All authors have read and approved the final manuscript.

## Conflict of Interest Statement

The authors declare that the research was conducted in the absence of any commercial or financial relationships that could be construed as a potential conflict of interest.

## Footnotes

1 http://www.combosyn.com

2 http://rsbweb.nih.gov/ij/

## Supplementary Material

FIGURE S1 Effect of normal glucose (G) and high glucose (HG) on Bcl-2, Bcl-XL, Bax, PARP, and cyt c protein expression in absence or presence of 5-FU in human colon cancer HT29 cells. Cells were cultured for ≥7 days in the presence of G and HG, incubated for 24 h before analysis with 25 and 50 μM 5-FU, then washed and lysed. The level of GAPDH, used as an housekeeping protein in total lysates, and the level of β-tubulin, used as an housekeeping protein in mitochondrial lysates, were used to check the equal protein loading. The figure is representative of two independent experiments.

FIGURE S2 Densitometry of Bcl-2, Bcl-XL, Bax, PARP, and cyt c protein expression in absence or presence of 5-FU in human colon cancer HT29 cells in G and HG conditions. The protein bands of two independent experiments have been quantified by densitometry and the values are expressed as arbitrary units. **(A)** For Bcl-2 measurements: ^∗∗^*p* < 0.001 and ^∗∗∗^*p* < 0.0001 25 and 50 μM, respectively, 5-FU in HT29 cells cultured in G vs. 0 μM 5-FU in HT29 cells cultured in G; ^••^*p* < 0.001 50 μM 5-FU in HT29 cells cultured in HG vs. 50 μM 5-FU in HT29 cells cultured in G. **(B)** For Bcl-XL measurements: ^∗∗^*p* < 0.001 25 and 50 μM, respectively, 5-FU in HT29 cells cultured in G vs. 0 μM 5-FU in HT29 cells cultured in G; ^§§^ *p* < 0.001 0 μM 5-FU in HT29 cells cultured in HG vs. 0 μM 5-FU in HT29 cells cultured in G; ^••^*p* < 0.01 25 and 50 μM 5-FU in HT29 cells cultured in HG vs. 25 and 50 μM 5-FU in HT29 cells cultured in G. **(C)** For Bax measurements: ^∗∗∗^*p* < 0.0001 50 μM 5-FU in HT29 cells cultured in G vs. 0 μM 5-FU in HT29 cells cultured in G; ^§^ *p* < 0.001 0 μM 5-FU in HT29 cells cultured in HG vs. 0 μM 5-FU in HT29 cells cultured in G; °*p* < 0.002 and ^∘∘^*p* < 0.001 25 and 50 μM, respectively, 5-FU in HT29 cells cultured in HG vs. 0 μM 5-FU X in HT29 cells cultured in HG; ^••^*p* < 0.001 50 μM 5-FU in HT29 cells cultured in HG vs. 50 μM 5-FU in HT29 cells cultured in G. **(D)** For Bax/Bcl-2 ratio measurements: ^∗^*p* < 0.001 and ^∗∗∗^*p* < 0.0001 25 and 50 μM, respectively, 5-FU in HT29 cells cultured in G vs. 0 μM 5-FU in HT29 cells cultured in G; ^••^*p* < 0.001 50 μM 5-FU in HT29 cells cultured in HG vs. 50 μM 5-FU in HT29 cells cultured in G. **(E)** For Bax/Bcl-XL ratio measurements: ^∗^*p* < 0.01 and ^∗∗∗^*p* < 0.001, respectively, 25 and or 50 μM 5-FU in HT29 cells cultured in G vs. 0 μM 5-FU in HT29 cells cultured in G; ^§^ *p* < 0.001 0 μM 5-FU in HT29 cells cultured in HG vs. 0 μM 5-FU in HT29 cells cultured in G; ^••^*p* < 0.001 and ^•••^*p* < 0.0001, respectively, 25 and or 50 μM 5-FU in HT29 cells cultured in HG vs. 25 and or 50 μM 5-FU in HT29 cells cultured in G. **(F)** For full length PARP measurements: ^∗∗^*p* < 0.001 and ^∗∗∗^*p* < 0.0001 25 and 50 μM, respectively, 5-FU in HT29 cells cultured in G vs. 0 μM 5-FU in HT29 cells cultured in G; ^••^*p* < 0.001 respectively, 25 and or 50 μM 5-FU in HT29 cells cultured in HG vs. 25 and or 50 μM 5-FU in HT29 cells cultured in G. **(H)** For cit cyt c measurements: ^∗∗∗^*p* < 0.0001 25 and 50 μM, respectively, 5-FU in HT29 cells cultured in G vs. 0 μM 5-FU in HT29 cells cultured in G; ^§^ *p* < 0.001 0 μM 5-FU in HT29 cells cultured in HG vs. 0 μM 5-FU in HT29 cells cultured in G; °*p* < 0.001 25 and 50 μM 5-FU in HT29 cells cultured in HG vs. 0 μM DOX in HT29 cells cultured in HG; ^••^*p* < 0.0001 25 and 50 μM 5-FU, respectively, in HT29 cells cultured in HG vs. 25 and 50 μM 5-FU, respectively, DOX in HT29 cells cultured in G. **(G,I)** For GAPDH and β-tubulin measurements: n.s.

FIGURE S3 Effect of normal glucose (G) and high glucose (HG) on Bcl-2, Bcl-XL, Bax, PARP, and cyt c protein expression in absence or presence of 5-FU (5-FU) in human colon cancer LOVO cells. Cells were cultured for ≥7 days in the presence of G and HG, incubated for 24 h before analysis with 25 and 50 μM 5-FU, then washed and lysed. The level of GAPDH, used as an housekeeping protein in total lysates, and the level of β-tubulin, used as an housekeeping protein in mitochondrial lysates, were used to check the equal protein loading. The figure is representative of three independent experiments.

FIGURE S4 Densitometry of Bcl-2, Bcl-XL, Bax, PARP, and cyt c protein expression in absence or presence of 5-FU in human colon cancer LOVO cells in G and HG conditions. The protein bands of two independent experiments have been quantified by densitometry and the values are expressed as arbitrary units. **(A)** For Bcl-2 measurements: ^∗^*p* < 0.01 25 and 50 μM, respectively, 5-FU in HT29 cells cultured in G vs. 0 μM DOX in HT29 cells cultured in G; ^••^*p* < 0.001 and ^•••^*p* < 0.0001 25 and 50 μM, respectively, 5-FU in HT29 cells cultured in HG vs. 25 and 50 μM, respectively, 5-FU in HT29 cells cultured in G. **(B)** For Bcl-XL measurements: ^∗∗∗^*p* < 0.0001 50 μM 5-FU in HT29 cells cultured in G vs. 0 μM 5-FU in HT29 cells cultured in G; ^§§^ *p* < 0.001 0 μM 5-FU in HT29 cells cultured in HG vs. 0 μM 5-FU in HT29 cells cultured in G; ?*p* < 0.001 and ^••^*p* < 0.0001 25 and 50 μM, respectively, 5-FU in HT29 cells cultured in HG vs. 25 and 50 μM, respectively, 5-FU in HT29 cells cultured in G. **(C)** For Bax measurements: ^∗∗∗^*p* < 0.0001 50 μM 5-FU in HT29 cells cultured in G vs. 0 μM 5-FU in HT29 cells cultured in G; ^•••^*p* < 0.0001 50 μM 5-FU in HT29 cells cultured in HG vs. 50 μM 5-FU in HT29 cells cultured in G. **(D)** For Bax/Bcl-2 ratio measurements: ^∗^*p* < 0.01 and ^∗∗∗^*p* < 0.0001 25 and 50 μM, respectively, 5-FU in HT29 cells cultured in G vs. 0 μM 5-FU in HT29 cells cultured in G; ^§^ *p* < 0.001 0 μM 5-FU in HT29 cells cultured in HG vs. 0 μM 5-FU in HT29 cells cultured in G; ?*p* < 0.01 and ^•••^*p* < 0.0001 25 and 50 μM, respectively, 5-FU in HT29 cells cultured in HG vs. 25 and 50 μM, respectively, 5-FU in HT29 cells cultured in G. **(E)** For Bax/Bcl-XL ratio measurements: ^∗^*p* < 0.01 and ^∗∗∗^*p* < 0.0001 25 and 50 μM, respectively, 5-FU in HT29 cells cultured in G vs. 0 μM 5-FU in HT29 cells cultured in G; ^§^ *p* < 0.001 0 μM 5-FU in HT29 cells cultured in HG vs. 0 μM 5-FU in HT29 cells cultured in G; ?*p* < 0.01 and ^•••^*p* < 0.0001 25 and 50 μM, respectively, 5-FU in HT29 cells cultured in HG vs. 25 and 50 μM, respectively, 5-FU in HT29 cells cultured in G. **(F)** For full length PARP measurements: ^∗∗^*p* < 0.001 and ^∗∗∗^*p* < 0.0001 25 and 50 μM, respectively, 5-FU in HT29 cells cultured in G vs. 0 μM 5-FU in HT29 cells cultured in G; ^••^*p* < 0.001 0 μM 525 and 50 μM, respectively, 5-FU in HT29 cells cultured in HG vs. 25 and 50 μM, respectively, 5-FU in HT29 cells cultured in G. **(H)** For cit cyt c measurements: ^∗∗^*p* < 0.001 and ^∗∗∗^*p* < 0.0001 25 and 50 μM, respectively, 5-FU in HT29 cells cultured in G vs. 0 μM 5-FU in HT29 cells cultured in G; °*p* < 0.001 25 and 50 μM, respectively, 5-FU in HT29 cells cultured in HG vs. 0 μM DOX in HT29 cells cultured in HG; ^••^*p* < 0.0001 25 and 50 μM, respectively, 5-FU in HT29 cells cultured in HG vs. 25 and 50 μM, respectively, 5-FU in HT29 cells cultured in G. **(G,I)** For GAPDH and β-tubulin measurements: n.s.
