# Supplementary figures and images for: Hyperglycemia Promotes Chemoresistance Through the Reduction of the Mitochondrial DNA Damage, the Bax/Bcl-2 and Bax/Bcl-XL Ratio, and the Cells in Sub-G1 Phase Due to Antitumoral Drugs Induced-Cytotoxicity in Human Colon Adenocarcinoma Cells
Source: Front Pharmacol. 2018 Aug 13;9:866. doi: 10.3389/fphar.2018.00866 (PMC6099160; doi:10.3389/fphar.2018.00866)

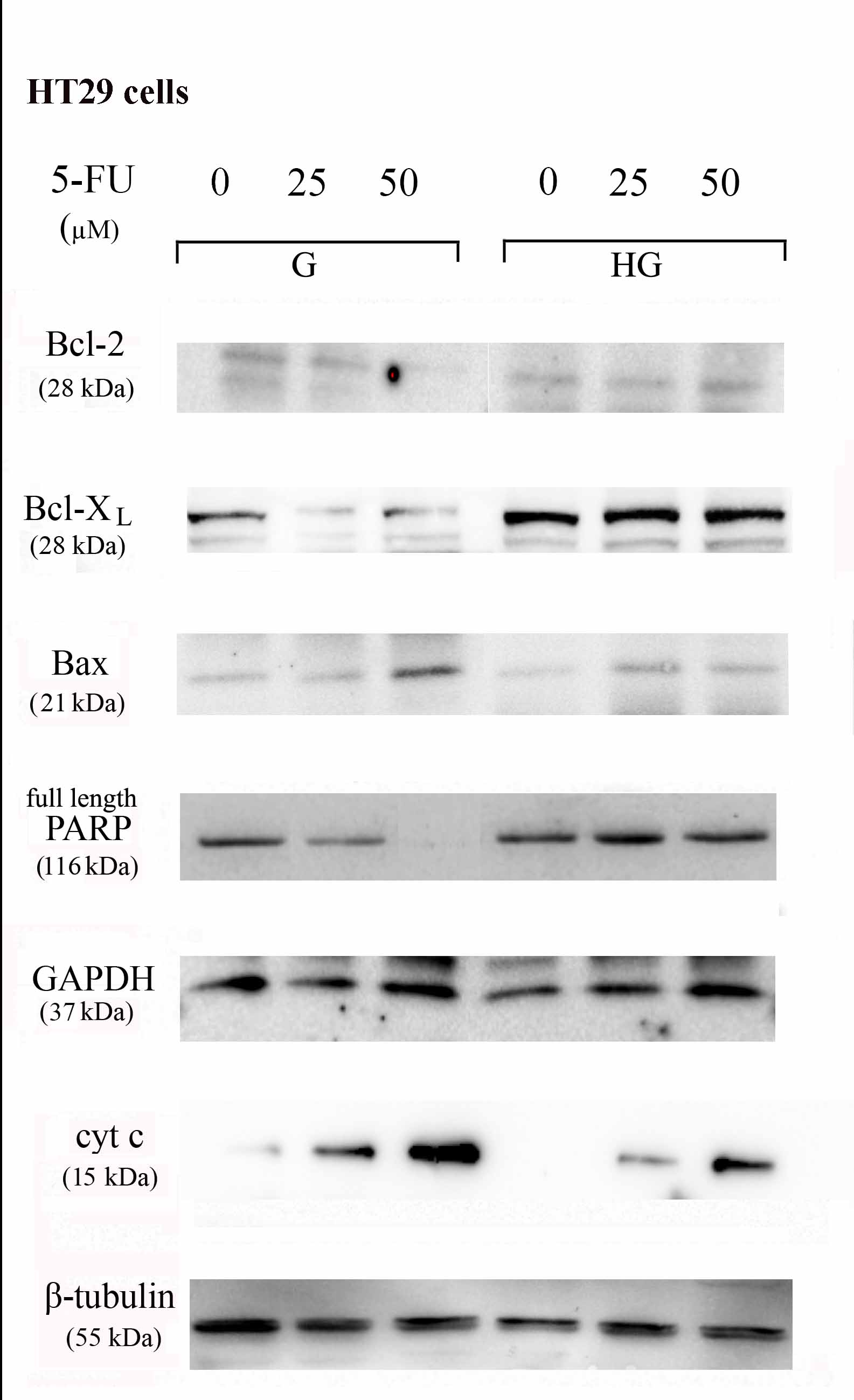

Supplement: FIGURE S1 — Effect of normal glucose (G) and high glucose (HG) on Bcl-2, Bcl-XL, Bax, PARP, and cyt c protein expression in absence or presence of 5-FU in human colon cancer HT29 cells. Cells were cultured for ≥7 days in the presence of G and HG, incubated for 24 h before analysis with 25 and 50 μM 5-FU, then washed and lysed. The level of GAPDH, used as an housekeeping protein in total lysates, and the level of β-tubulin, used as an housekeeping protein in mitochondrial lysates, were used to check the equal protein loading. The figure is representative of two independent experiments. [file Image_1.JPEG]

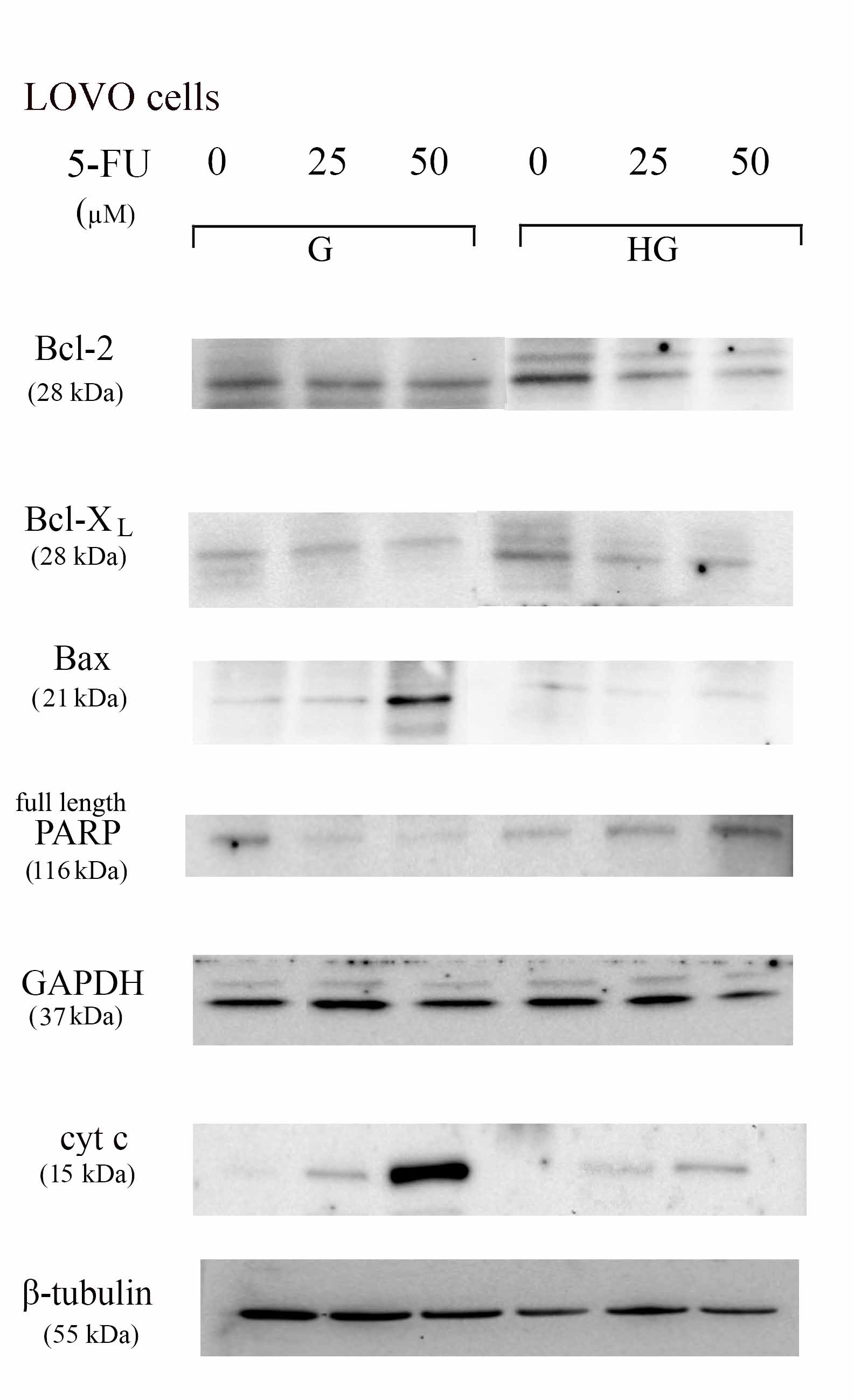

Supplement: FIGURE S3 — Effect of normal glucose (G) and high glucose (HG) on Bcl-2, Bcl-XL, Bax, PARP, and cyt c protein expression in absence or presence of 5-FU (5-FU) in human colon cancer LOVO cells. Cells were cultured for ≥7 days in the presence of G and HG, incubated for 24 h before analysis with 25 and 50 μM 5-FU, then washed and lysed. The level of GAPDH, used as an housekeeping protein in total lysates, and the level of β-tubulin, used as an housekeeping protein in mitochondrial lysates, were used to check the equal protein loading. The figure is representative of three independent experiments. [file Image_3.JPEG]

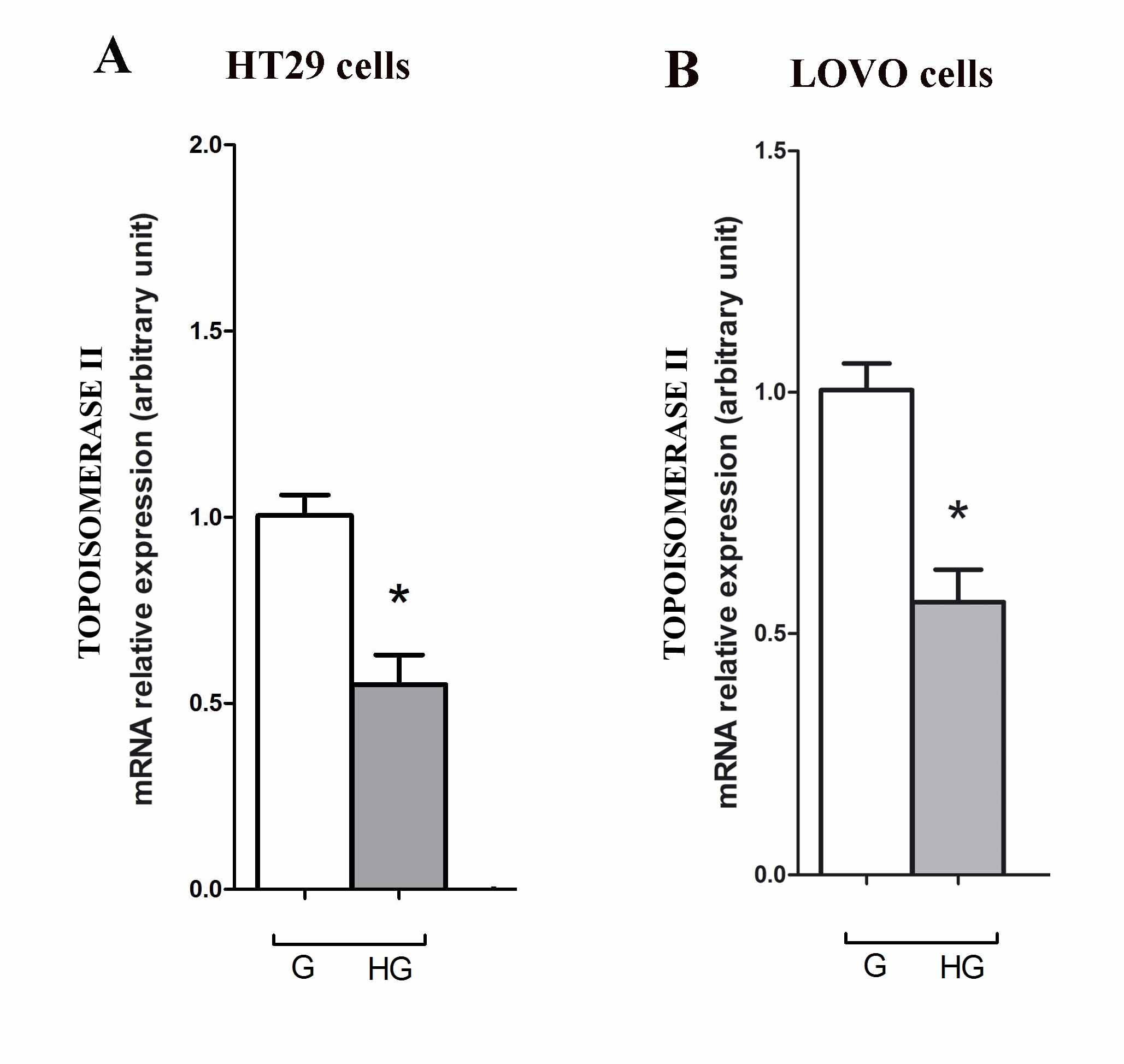

Supplement: Supplementary file 5 [file Data_Sheet_1.ZIP › FIG 13 MOD.jpg]

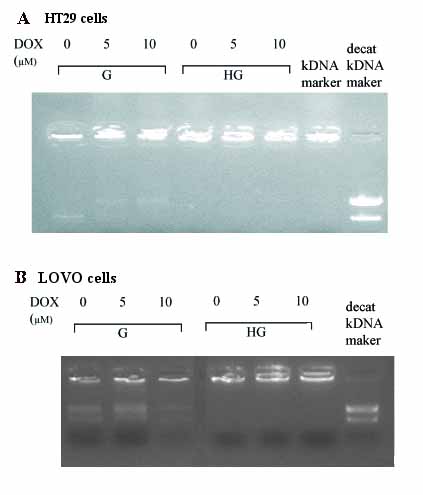

Supplement: Supplementary file 5 [file Data_Sheet_1.ZIP › FIG 14.jpg]

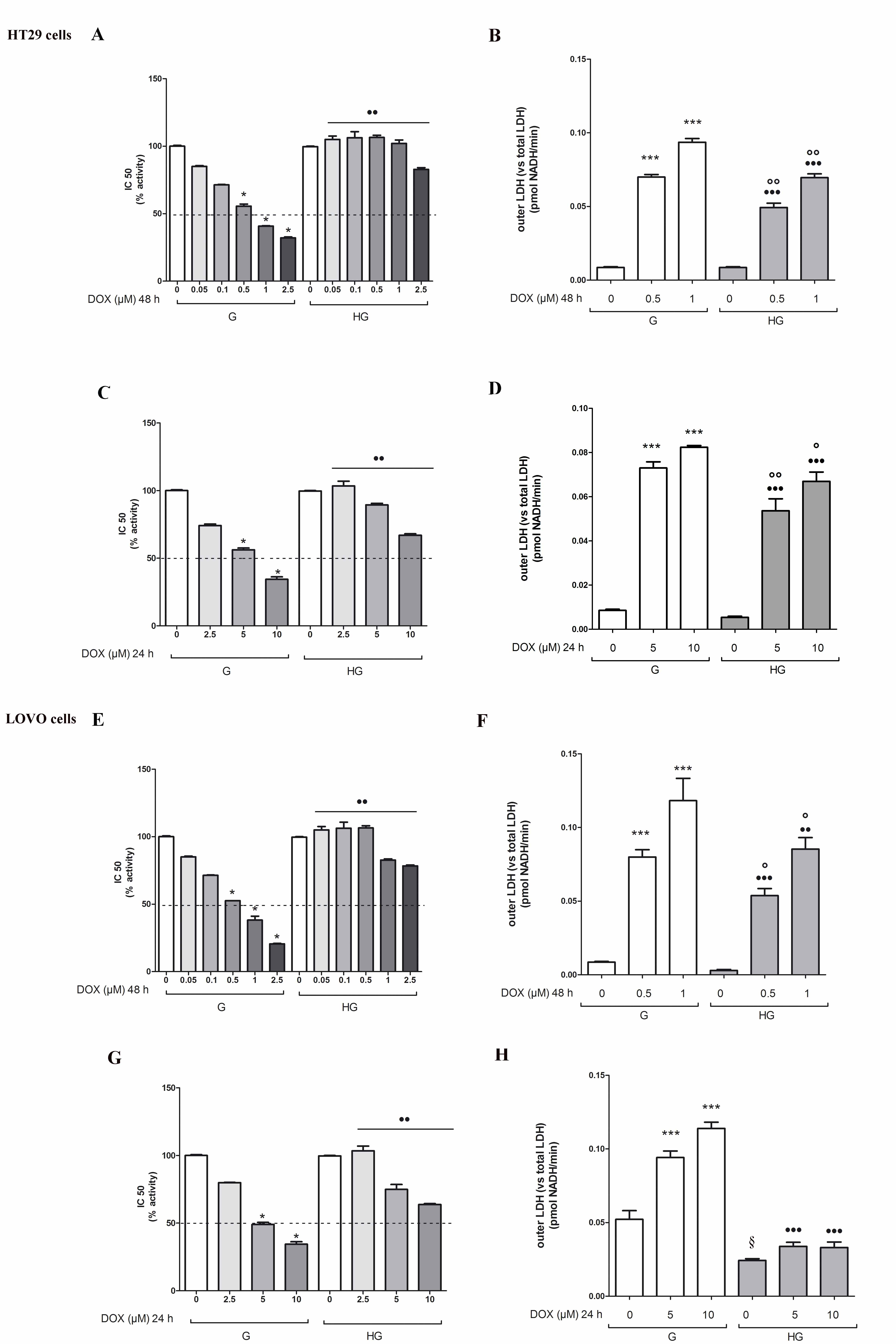

Supplement: Supplementary file 5 [file Data_Sheet_1.ZIP › FIG 1.jpg]

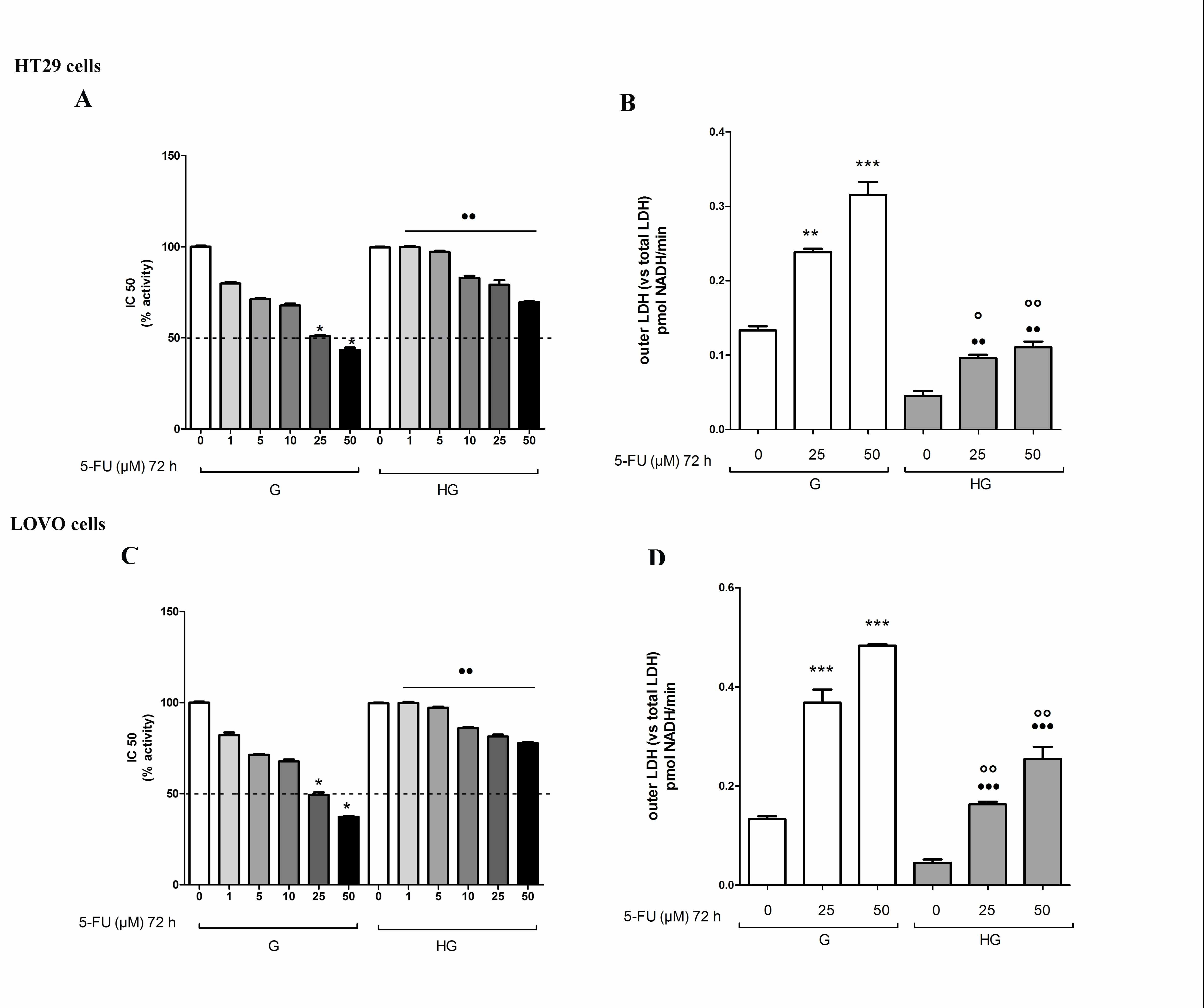

Supplement: Supplementary file 5 [file Data_Sheet_1.ZIP › Fig 2.jpg]

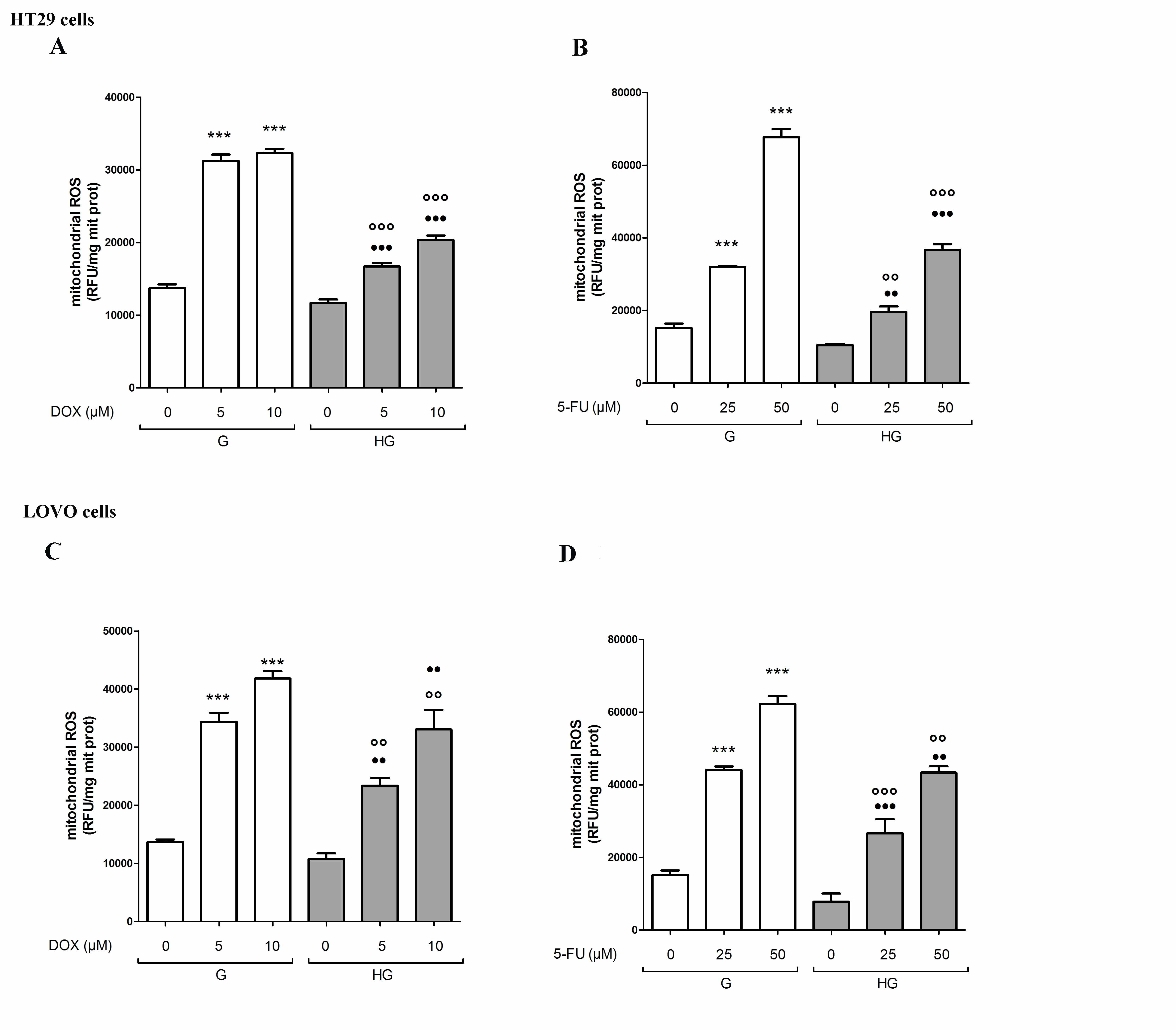

Supplement: Supplementary file 5 [file Data_Sheet_1.ZIP › FIG 3.jpg]

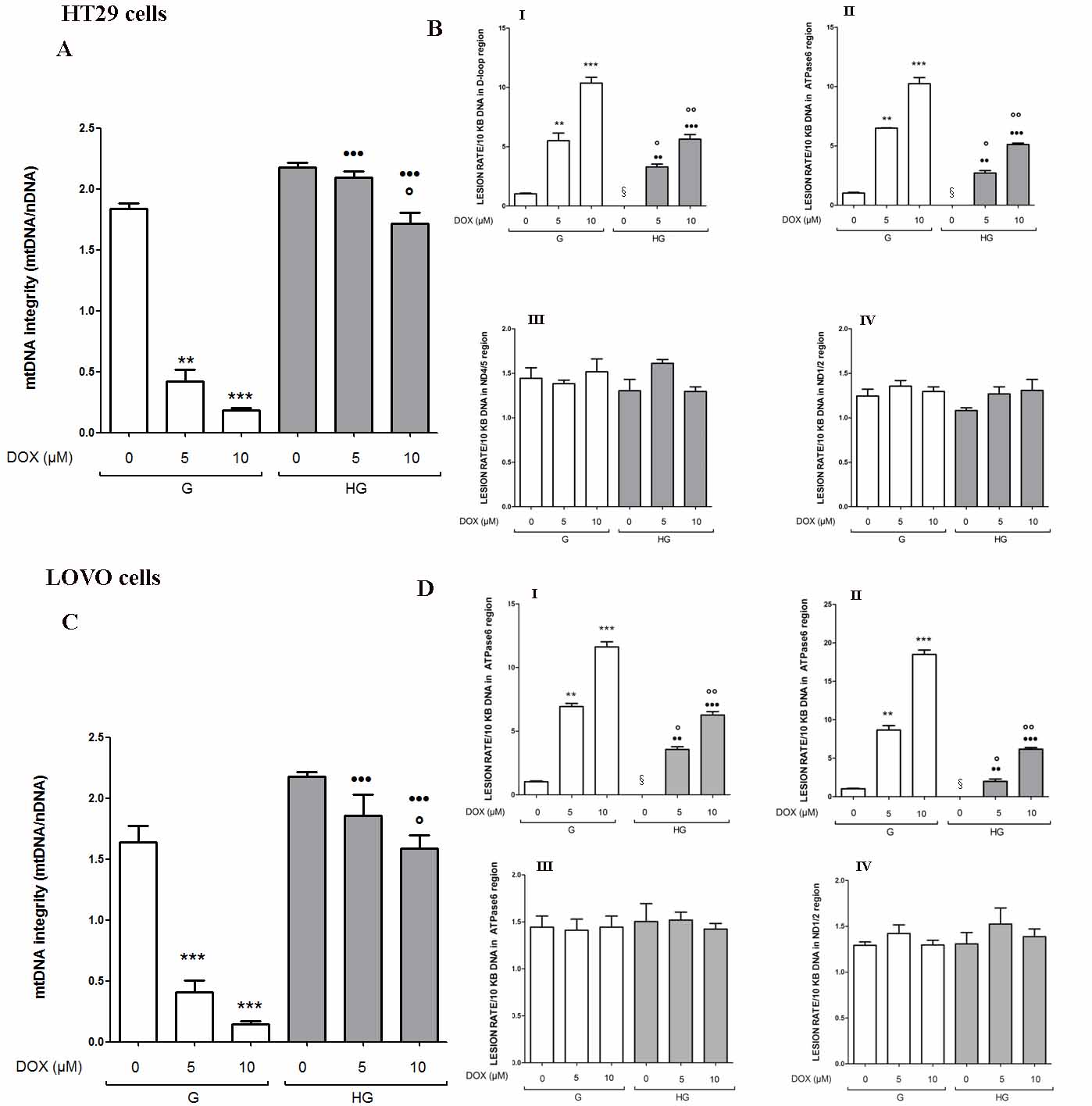

Supplement: Supplementary file 5 [file Data_Sheet_1.ZIP › FIG 4.jpg]

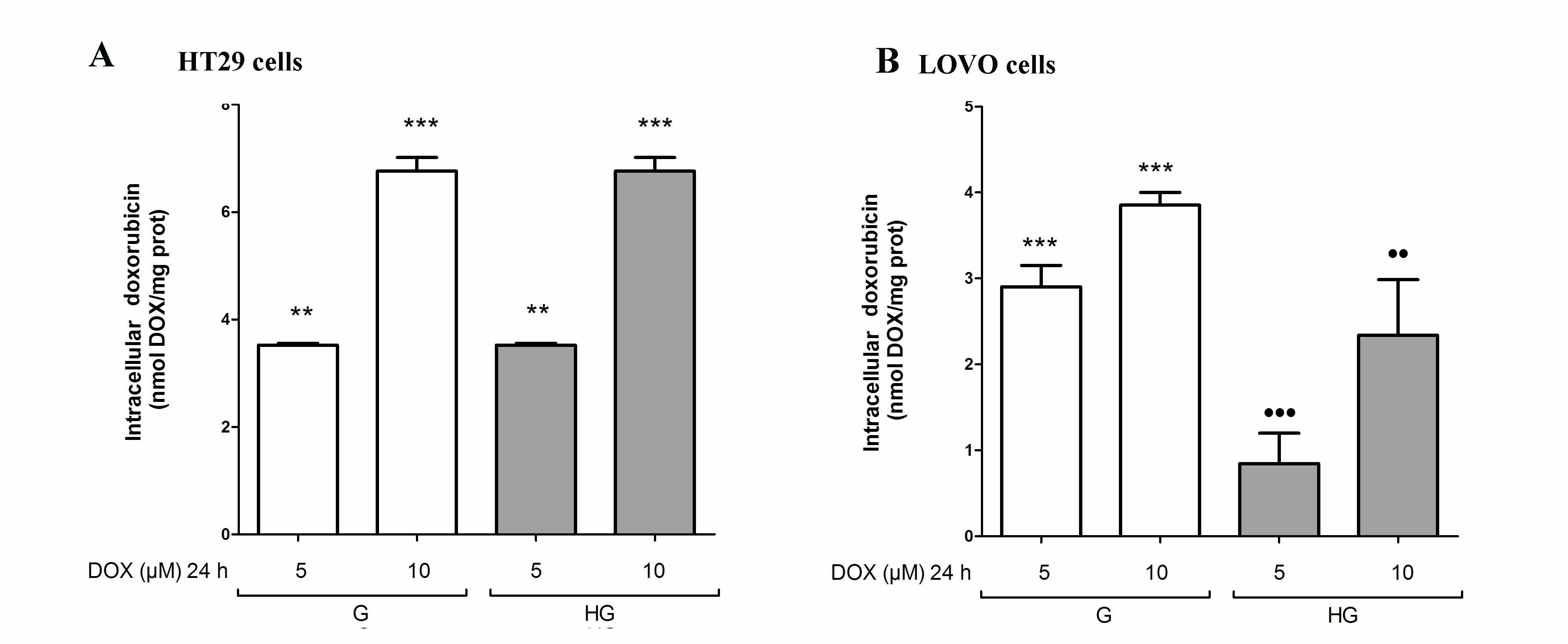

Supplement: Supplementary file 5 [file Data_Sheet_1.ZIP › FIG 5.jpg]

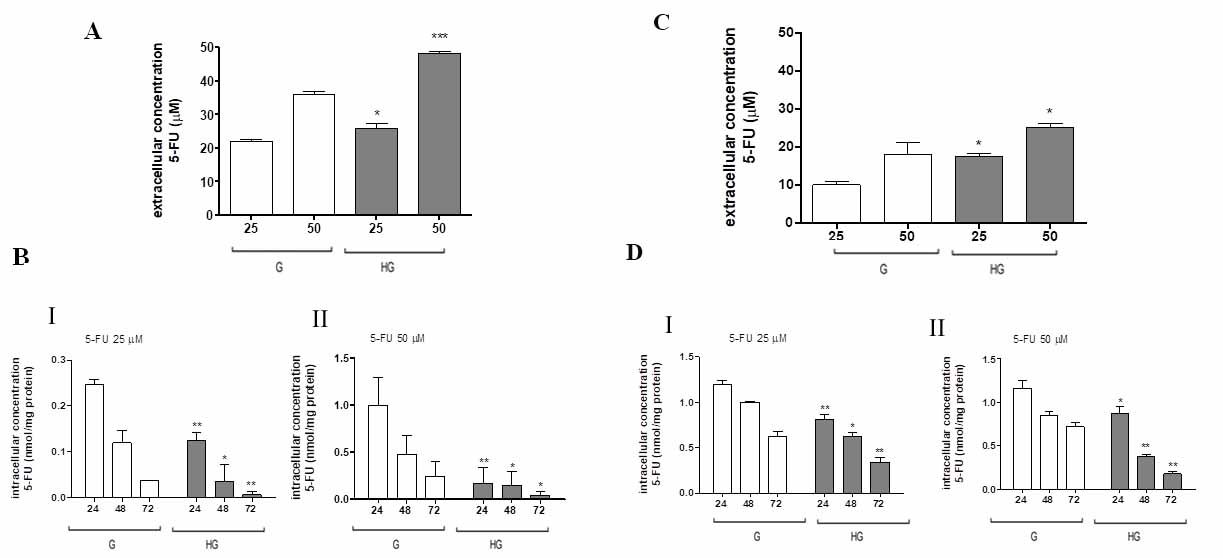

Supplement: Supplementary file 5 [file Data_Sheet_1.ZIP › FIG 6 MOD.jpg]

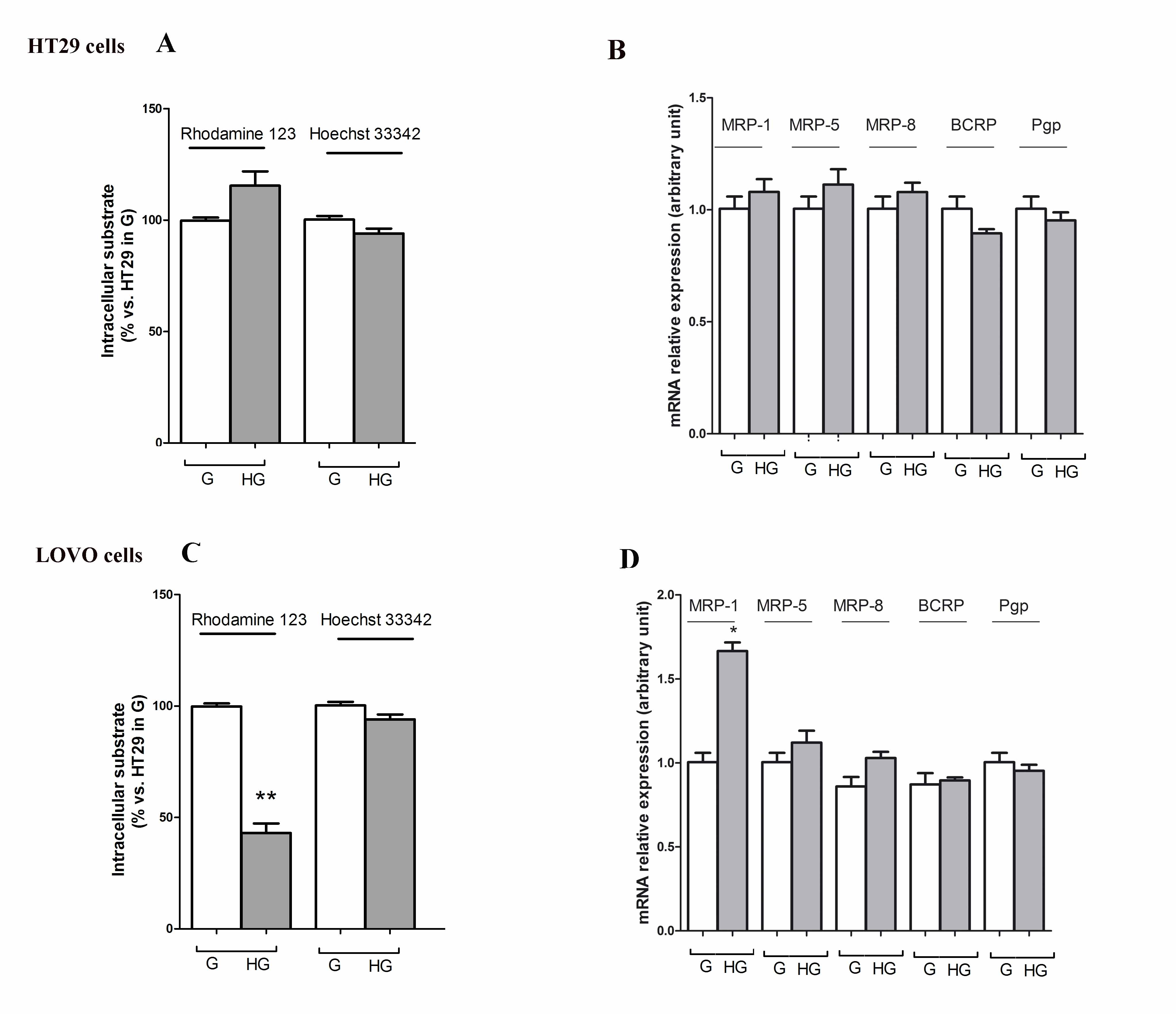

Supplement: Supplementary file 5 [file Data_Sheet_1.ZIP › FIG 7.jpg]

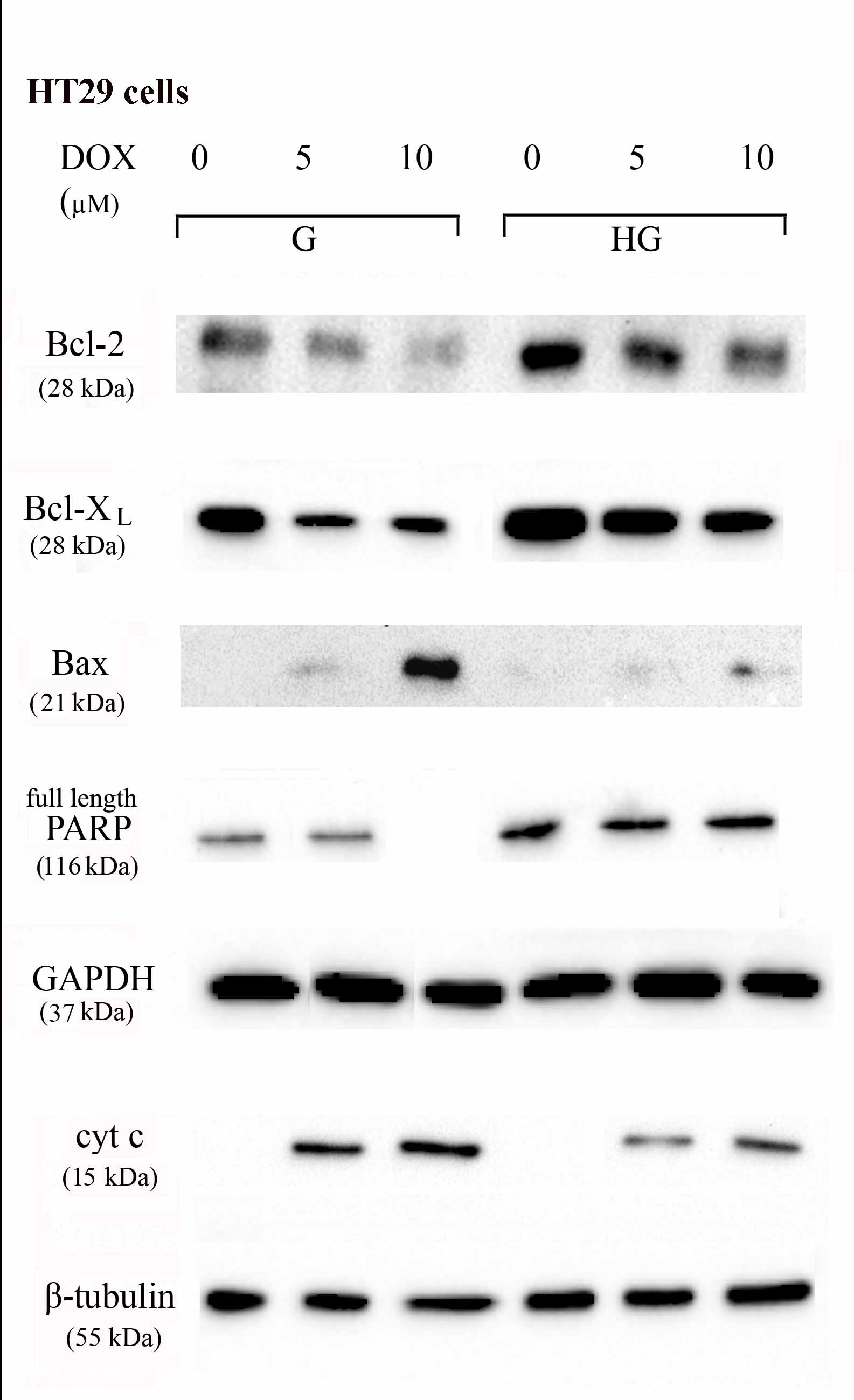

Supplement: Supplementary file 5 [file Data_Sheet_1.ZIP › FIG 8 MOD.jpg]

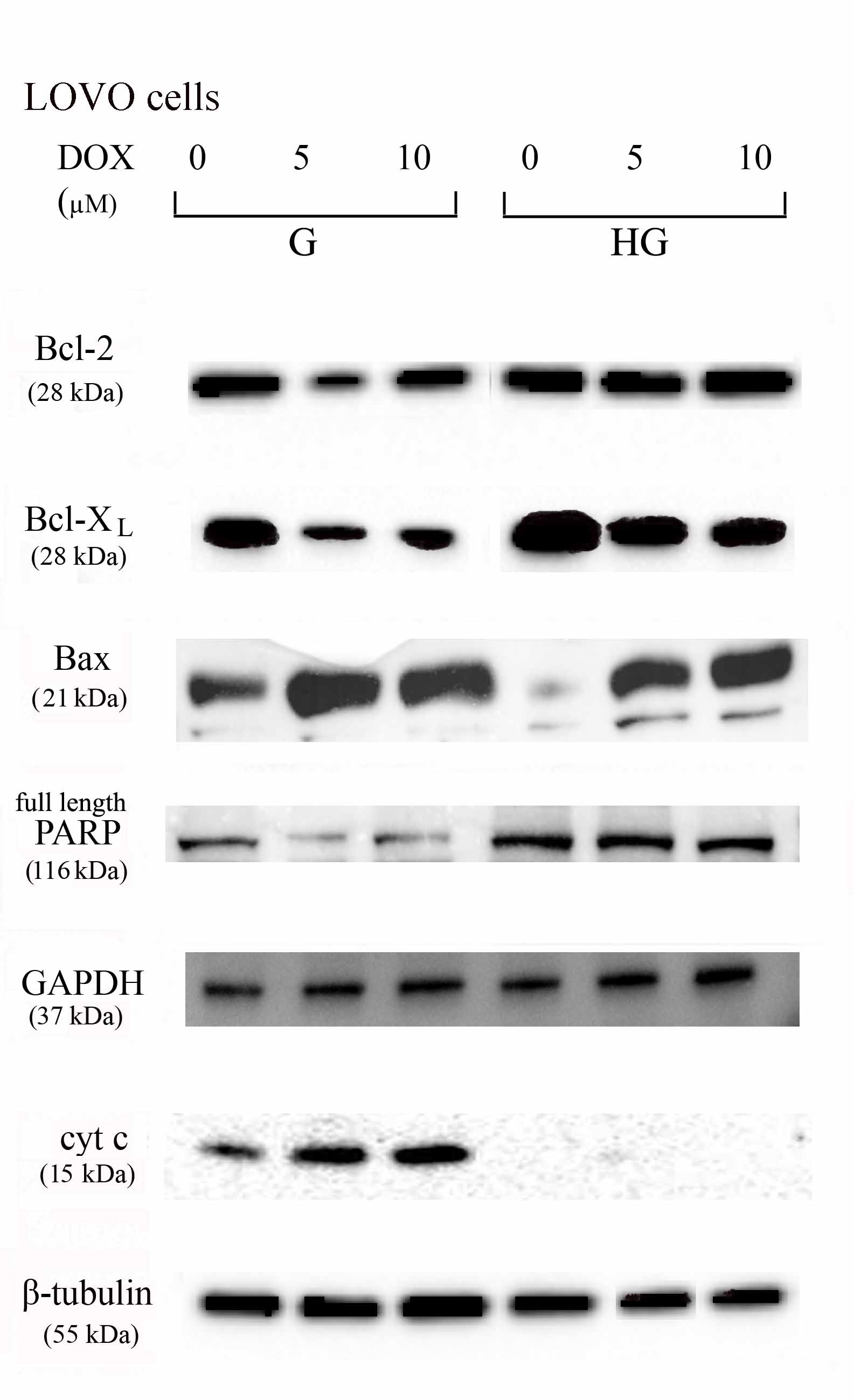

Supplement: Supplementary file 5 [file Data_Sheet_1.ZIP › FIG 10 MOD.jpg]

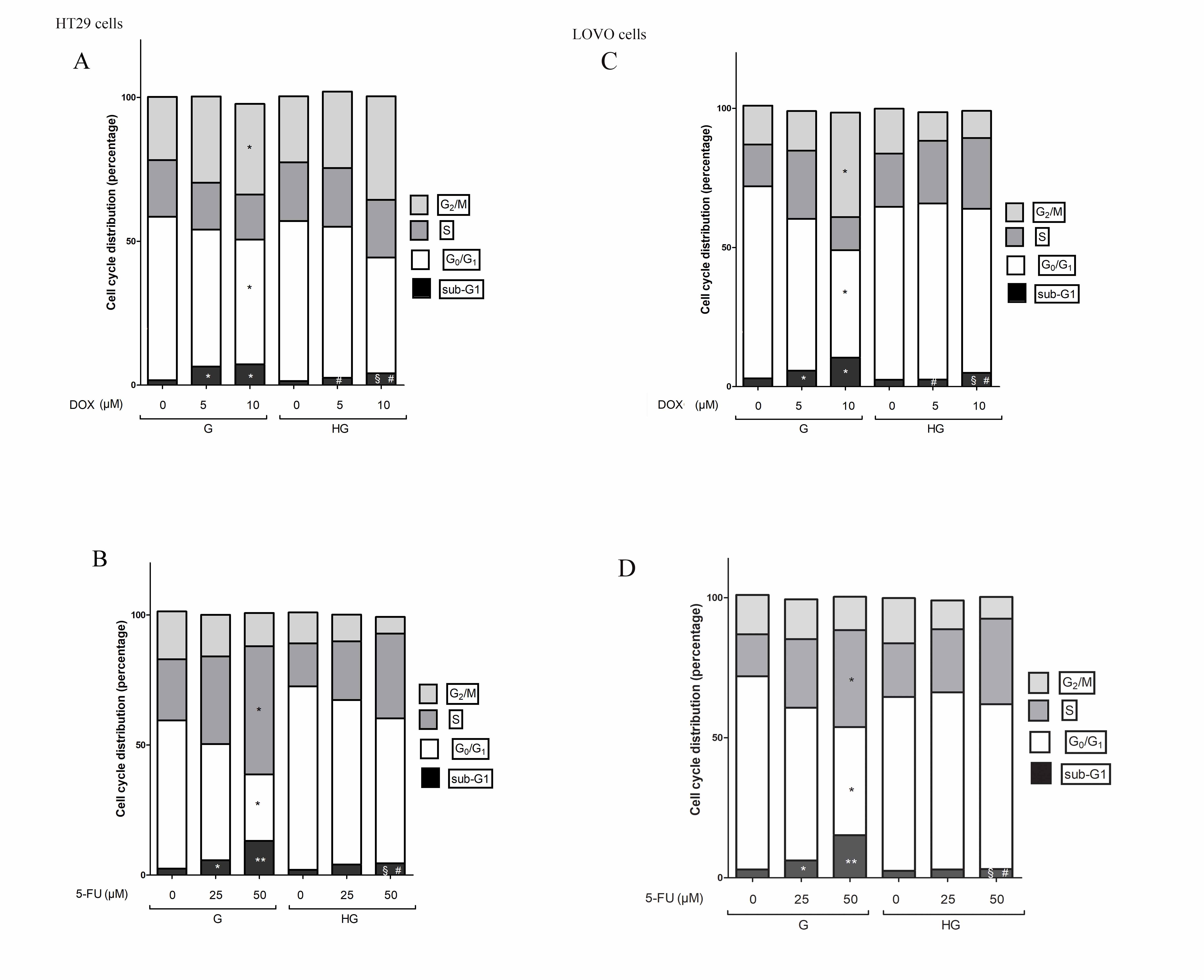

Supplement: Supplementary file 5 [file Data_Sheet_1.ZIP › FIG 12_MOD.jpg]
